# Assessing ex situ genetic and ecogeographic conservation in a threatened but widespread oak after range‐wide collecting effort

**DOI:** 10.1111/eva.13391

**Published:** 2022-05-31

**Authors:** Bethany A. Zumwalde, Bailie Fredlock, Emily Beckman Bruns, Drew Duckett, Ross A. McCauley, Emma Suzuki Spence, Sean Hoban

**Affiliations:** ^1^ Center for Tree Science The Morton Arboretum Lisle Illinois USA; ^2^ 33626 Department of Biology University of Florida Gainesville Florida USA; ^3^ Global Tree Conservation The Morton Arboretum Lisle Illinois USA; ^4^ 2647 Department of Evolution, Ecology and Organismal Biology The Ohio State University Columbus Ohio USA; ^5^ Department of Biology Fort Lewis College Durango Colorado USA; ^6^ The Field Museum Chicago Illinois USA

**Keywords:** alleles, conservation planning, IUCN Red List, metacollection, microsatellites, sampling

## Abstract

Although the genetic diversity and structure of in situ populations has been investigated in thousands of studies, the genetic composition of ex situ plant populations has rarely been studied. A better understanding of how much genetic diversity is conserved ex situ, how it is distributed among locations (e.g., botanic gardens), and what minimum sample sizes are needed is necessary to improve conservation outcomes. Here we address these issues in a threatened desert oak species, *Quercus havardii* Rydb. We assess the genetic, geographic, and ecological representation of 290 plants from eight ex situ locations, relative to 667 wild individuals from 35 in situ locations. We also leverage a recent dataset of >3000 samples from 11 other threatened plants to directly compare the degree of genetic conservation for species that differ in geographic range size. We found that a majority of *Q*. *havardii* genetic diversity is conserved; one of its geographic regions is significantly better conserved than the other; genetic diversity conservation of this widespread species is lower than documented for the 11 rarer taxa; genetic diversity within each garden is strongly correlated to the number of plants and number of source populations; and measures of geographic and ecological conservation (i.e., percent area and percent of ecoregions represented) were typically lower than the direct assessment of genetic diversity (i.e., percent alleles). This information will inform future seed sampling expeditions to ensure that the intraspecific diversity of threatened plants can be effectively conserved.

## 1 INTRODUCTION

Decades of population genetic studies have revealed the intrinsic (e.g., traits, geographic range size) and extrinsic (e.g., habitat quality, anthropogenic fragmentation) drivers of genetic diversity and structure in wild populations of plants and animals (Aguilar et al., 2008; Allendorf, 2017; Loveless & Hamrick, 1984). The genetic diversity and structure of highly managed and captive‐bred animal populations, as well as seed banks of important crops and their wild relatives, have also been studied (e.g., Ogden et al., 2020; Singh et al., 2019). However, there is remarkably little knowledge of genetic diversity and structure in ex situ populations (such as botanic gardens, arboreta, and seed banks) for most plant species, even though thousands of botanic gardens globally hold over 100,000 plant species (Mounce et al., 2017). Relatively few studies have quantified genetic diversity patterns ex situ or compared ex situ and in situ populations (Christe et al., 2014; McGlaughlin et al., 2015; Namoff et al., 2010), while even fewer have sought to understand the drivers of these patterns (e.g., species traits, comprehensiveness of collection efforts, botanic garden management practices; see Griffith et al., 2017; Hoban, 2019; Hoban, Callicrate, et al., 2020; Hoban & Schlarbaum, 2014). This is a major gap in the field of molecular ecology and conservation genetics.

Safeguarding species and populations ex situ is an essential component of conservation programs, especially when in situ threats are high (Oldfield, 2009; Westwood et al., 2021). Ex situ plant collections can be composed of seed banks, tissue culture, and frozen tissue or embryos, which require relatively little space, as well as living mature plants (i.e., ‘living collections’), which take up orders of magnitude more space and have higher costs. Living collections offer some advantages over seed collections. They can help produce seed or cloned individuals for restoration or reintroduction if in situ populations are lost, increase public understanding and appreciation of biodiversity, allow scientific study of rare species, and provide genetic and functional trait diversity for breeding programs (Cavender et al., 2015; Heywood, 2017). Living collections are especially important for species that produce recalcitrant seeds (i.e., those that do not tolerate desiccation for storage in conventional seed banks). Approximately 8% of all plants (and 27% of threatened plants) are recalcitrant (Wyse & Dickie, 2017; Wyse et al., 2018). The space requirements and monetary cost of living collections mean that it is particularly important to evaluate and optimize ex situ genetic diversity.

Ensuring high genetic and trait diversity in ex situ collections is important for long‐term persistence of a species under environmental change (e.g., climate change, new pests, and diseases). Botanic gardens can provide seed and plant material for ecological restoration, and restoration success can be influenced by genetic diversity (Breed et al., 2019). It is also increasingly apparent that genetic diversity, especially in trees and other keystone species, contributes to community structure and ecosystem resilience (Raffard et al., 2018; Reusch et al., 2005; Stange et al., 2020), as well as nature's contributions to people (Des Roches et al., 2021). However, living ex situ collections often have few individuals and/or were collected from only a few wild sources (e.g., many species have fewer than 50 plants in collections globally (Beckman et al., 2019; Hoban & Way, 2016; Maunder et al., 2001). Ex situ populations thus may have insufficient genetic diversity for species’ long‐term survival.

Ideally, most of the alleles that exist in situ should be protected ex situ, preferably in multiple locations for safekeeping, which later could be used for applications such as plant reintroductions or breeding programs (Brown & Marshall, 1995; Lawrence et al., 1995; Lockwood et al., 2007). Genetic markers, applied to tissue samples from ex situ collections and from wild populations, are an increasingly accessible and affordable way to assess genetic diversity and structure ex situ, as shown by several recent efforts. For example, Griffith et al. (2015) showed that 205 ex situ plants captured 78% of the alleles present in two wild populations of *Zamia decumbens* (Zamiaceae), while Hoban, Callicrate, et al. (2020) quantified how ex situ sampling strategies can be improved for 11 plant taxa across five genera. Such case studies in species with differing life‐history characteristics help to establish “rules” for how genetic diversity ex situ is impacted by collection size, species’ biological traits, and other factors such as geographic range size in situ (Griffith et al., 2017; Hoban, 2019; Hoban, Bruford, et al., 2020; Hoban & Strand, 2015). While the aforementioned studies are building such knowledge for rare, range‐restricted species, we are aware of no similar studies for species that are geographically widespread but still threatened. Predictions from models suggest that species with larger population sizes, more populations, and geographically disconnected populations will need more ex situ individuals in conservation collections to sufficiently preserve in situ genetic diversity (Brown & Hardner, 2000; Hoban, 2019; Hoban & Schlarbaum, 2014).

Ex situ collections should also represent geographic and ecological variation across a species range, which may help capture adaptive variation (Brown & Hardner, 2000; Guerrant et al., 2004). Ecological and geographic coverage is much easier to measure and may be an effective proxy for genetic diversity because genetic diversity typically increases with geographic (Alsos et al., 2012; Hanson et al., 2017) and environmental distance (Di Santo & Hamilton, 2020; Wang & Bradburd, 2014). Genetic diversity assessments still require large numbers of samples and specialized equipment and laboratory work; it is infeasible to collect population‐level genetic data to optimize collection strategies for the approximately 350,000 plant taxa that exist. Khoury et al. (2019) suggest that the percentage of a species’ geographic range represented by plants in ex situ collections can be a “pragmatic estimate of the comprehensiveness of conservation of the genetic diversity.” Measuring geographic coverage can help identify which species are sufficiently conserved, and prioritize among those that most need additional conservation effort. Such an approach has rarely been applied outside crop wild relatives (Khoury et al., 2020; Vinceti et al., 2013; though see Beckman et al., 2019), nor has this approach been directly compared to genetic assessments.

To address these major knowledge gaps, we assess patterns of genetic diversity within and among eight botanic gardens (containing 290 individuals), compared to in situ populations (667 individuals) of shinnery oak (*Quercus havardii* Rydb.), an uncommon but wide‐ranging shrub species with recalcitrant seeds. We also assess geographic and ecological proxies of genetic diversity conservation. This is, to our knowledge, the first extensive genetic analysis of ex situ collections of a widespread but uncommon species; previous work has mostly focused on highly rare species (Griffith et al., 2010, 2015; Hoban, Callicrate, et al., 2020; Namoff et al., 2010). One may predict that ex situ populations (we will use the term “ex situ population” herein to refer to sets of plants at different gardens, as others have previously; see Schaal & Leverich, 2004) of the widespread *Q*. *havardii* will have lower genetic diversity than collections of rarer, small‐ranged species, based on simulations demonstrating that range size and gene flow can impact genetic diversity in sampled seed collected for ex situ collections (Hoban, 2019; Hoban & Schlarbaum, 2014). However, ex situ collections of *Q*. *havardii* are large (290 seedlings collected and used for this study while Beckman et al., 2019 found that “the majority of U.S. oak species are represented by fewer than 150 plants in ex situ collections”) and were sampled using best practice recommendations (i.e., many maternal plants spread out in populations across much of the geographic range; see details of seed collection in Methods). Therefore, levels of genetic diversity of *Q*. *havardii* may exceed levels conserved for previously studied rarer taxa. To make a comparison between *Q*. *havardii* and rarer species, we use a recently published dataset of >3000 individuals of 11 threatened species (all less common than *Q*.* havardii*), and we apply the same molecular analysis techniques (Hoban, Callicrate, et al., 2020). We have four aims in this study:

1. Quantify the percent of the extant in situ genetic diversity of *Q*. *havardii* that is conserved ex situ, and calculate the minimum number of sampled individuals needed for ex situ conservation of 95% of the known species’ alleles.
2. Compare the percent of genetic diversity conserved and the minimum sampling needed (from aim 1) for this widespread species to values recently documented for 11 rarer species.
3. Quantify genetic diversity and structure within each of eight garden populations of *Q*. *havardii* and determine if genetic diversity within a garden is a function of the number of plants.
4. Compare the percent of genetic diversity conserved to two non‐genetic measures of ex situ conservation: percent of geographic range and percent of distinct ecological regions from which seed was sampled.

## 2 MATERIALS AND METHODS

### 2.1 Study species

*Quercus havardii* is currently listed as Endangered on the IUCN Red List due to ongoing decline in population size and increasing fragmentation and habitat loss resulting from human activities (e.g., changes in land use for grazing or oil and gas development, and deliberate eradication by landowners due to the poisonous effects on livestock and/or competition with crops for water; Kenny et al., 2020). *Quercus havardii* is typically restricted to deep sand dunes and sandy grasslands, an unusual habitat for oaks (Peterson & Boyd, 2000). Projected climate change resulting in a hotter, drier Southwest United States could challenge the persistence of this species (Beckman et al., 2019).

**FIGURE 1:**
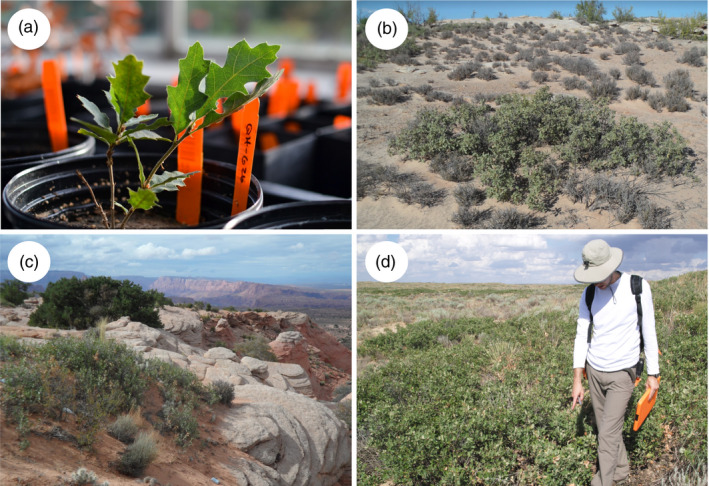
*Quercus havardii* ex situ at The Morton Arboretum (a); two example habitats from western in situ populations from the species' disjunct distribution (b and c); and an example from an in situ eastern population (d)

Although uncommon and restricted to a very specific habitat, *Q*. *havardii* is an ecologically important species where it does occur despite its diminutive height (0.2–1 m). Its large seeds (i.e., acorns) are an important food resource for wildlife. Notably, this species also provides habitat for the lesser prairie chicken (*Tympanuchus cupido*) and the dunes sagebrush lizard (*Sceloporus arenicolus*), both listed as Vulnerable on the IUCN Red List and continuing to decline (Boyd & Bidwell, 2001). Its extensive root system can be up to 10 m deep, which can help stabilize sand dunes (Nellessen, 2004; Peterson & Boyd, 2000). One individual may consist of many short stems in a dense clump from one to a dozen or more meters across (Figure 1). *Quercus havardii* is wind pollinated, with seeds that are presumably dispersed by rodents, gravity, and water. Many oaks show masting behavior (periodic years of a high number of seeds produced, e.g., every 3 or 5 years), though detailed observation has not been made for this species.

**FIGURE 2:**
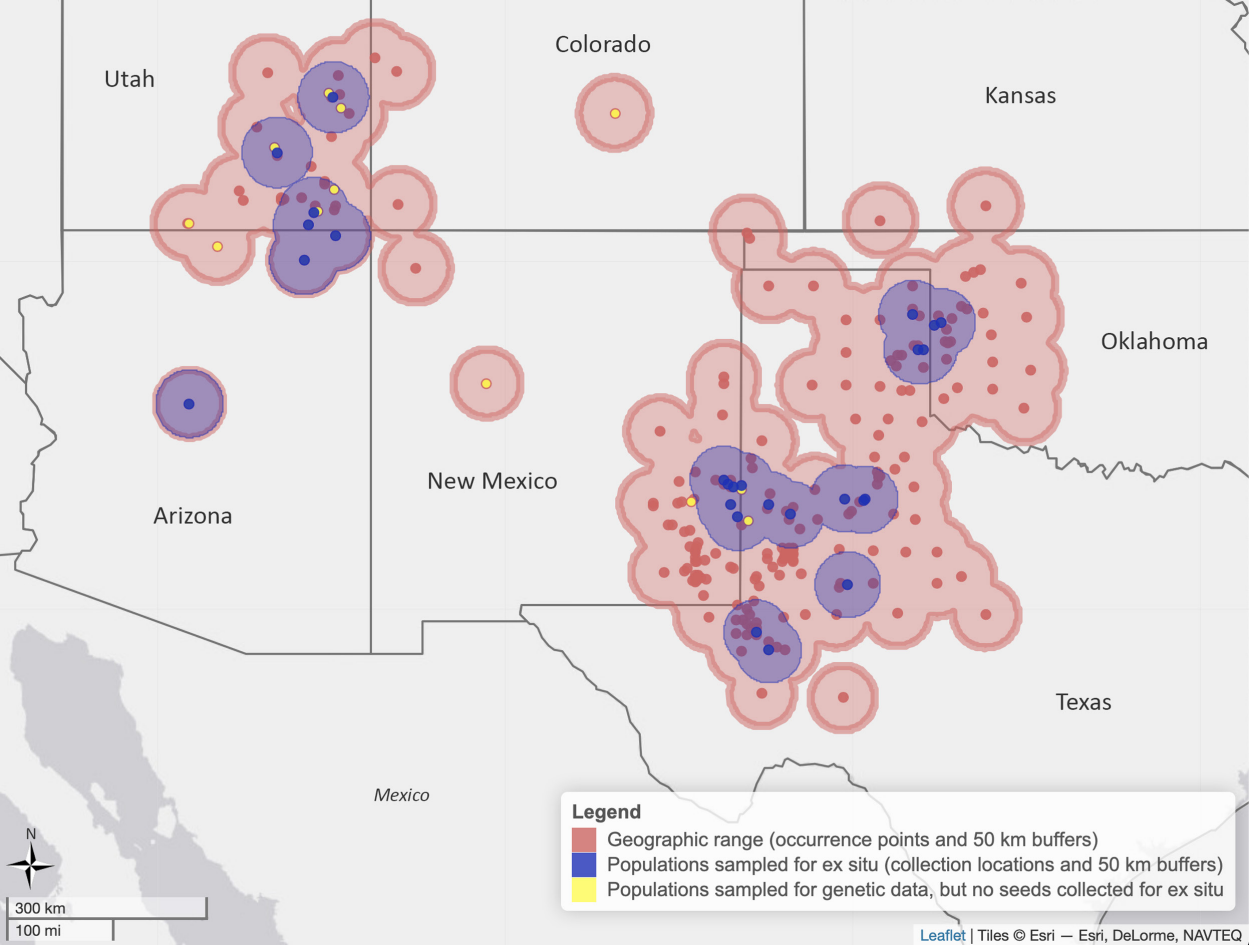
Map representation of the geographic range of *Quercus havardii* and populations that have been sampled for seed ex situ conservation

We note that *Q*. *havardii* has a disjunct range (Tucker, 1970), with a western group of populations in southern Utah, northern Arizona, and northwestern New Mexico, and an eastern group of populations in eastern New Mexico, Texas, and southern Oklahoma (Figure 2). The evolutionary history of the group and age of the disjunction is under ongoing investigation (McCauley et al., 2012; Zumwalde et al., 2021). Here, we treat all populations as one species, as it was treated in the latest IUCN Red List assessment (Jerome et al., 2017), while acknowledging these may constitute subspecies or distinct species.

### 2.2 Ex situ tissue collection

Prior to 2016, to our knowledge, only one botanic garden maintained *Quercus havardii* in its living collections. To help conserve this species, a large seed collection effort took place in 2016 as part of the US Forest Service− American Public Gardens Association Tree Gene Conservation Partnership (Hoban & Duckett, 2016). The focus of this program is to establish genetically diverse living gene banks of US threatened tree species by collecting seeds from across each species’ native range and then distributing the seeds to public gardens for safeguarding (https://www.publicgardens.org/programs/plant‐collections‐network/tree‐gene‐conservation‐partnership). Following best practices to maximize genetic diversity (see Hoban & Schlarbaum, 2014; Maschinski et al., 2019), few seeds per maternal plant were sampled (Figure S1; Table S1), while visiting as many populations as possible across the range. This collecting effort resulted in 1751 seeds from 30 populations from 67 maternal lines (e.g. mother plants) or accessions across the geographic range of *Q*. *havardii* (Hoban & Duckett, 2016), though numerous seeds were desiccated, immature, infested with weevils, or did not germinate. Note that seeds from a given maternal line will be at least half‐sibling relatives. These seeds collected for ex situ conservation were the offspring of the individuals used in the in situ population genetic study described in the next section. Seeds were distributed to other botanic gardens and sown in 2017 and 2018. Once seedlings had produced several leaves, leaves from 290 seedlings from 66 maternal trees representing 26 wild populations were collected from the botanical gardens (Figure S1; Table S1).

### 2.3 In situ tissue collection

During the seed collection described above, a total of 667 mature trees were sampled (leaf samples) from 36 populations of georeferenced locations in the summer of 2016 for analyses of in situ genetic diversity. A detailed analysis of in situ genetic structure can be found in Zumwalde et al. (2021). Populations selected to represent the geographic range of *Q*. *havardii* were chosen by consulting public and private land managers, the Global Biodiversity Information Facility (GBIF) and SEINET, and suggestions from the International Oak Society. One voucher specimen per population was deposited at each of the following herbaria: MOR, FLD, and NAVA (herbarium acronyms follow Holmgren & Holmgren, 1991). In most locations, *Q*. *havardii* is the only oak species present due to its highly specific habitat; thus, the potential for hybridization is likely low in these populations.

### 2.4 Molecular methods

DNA extraction was performed from approximately 0.035–0.060 grams of leaf material using E.Z.N.A. Plant DNA DS kits (Omega Bio‐tek, Inc.) with small modifications (Methods S1). DNA was quantified using a NanoDrop One spectrophotometer (Thermo Scientific) and diluted to approximately 10 ng/ul. Eleven microsatellite loci were chosen from several other oak species (Table S2). These loci are not known to be associated with functional genes and are extremely unlikely to be linked to each other, considering there are 12 oak chromosomes (Plomion et al., 2018) and linkage disequilibrium in wind‐pollinated forest trees is generally low (Neale & Kremer, 2011). Regardless, we calculated the Agapow and Burt (2001) index of association, $\underline{r}$_d_, for each pair of loci (Figure S2). Four positive and one negative control (water) were included on each 96‐well plate. PCR product was verified on electrophoresis gels and then prepared for DNA fragment analysis using Genescan LIZ 600 size standard (Thermo Fisher). Fragment analysis was performed on an ABI3730 at The Field Museum of Natural History (Chicago, IL, USA). Note that in situ individuals were primarily genotyped in 2018 and ex situ individuals in 2019, but all equipment and reagents were identical and allele calls were compatible, so there are unlikely to be any “batch effects” (Goh et al., 2017; Leek et al., 2010). Fragment sizes were analyzed using Geneious v.10.2.3 (Biomatters). Micro‐Checker 2.2.3 (Oosterhout et al., 2004) and INEST v.2.2 (Chybicki & Burczyk, 2008) were used to check for null alleles across loci for the in situ dataset (Table S3). We did not check for null alleles in the ex situ dataset because we expect high departures from Hardy‐Weinberg assumptions (e.g., there are numerous close relatives in the ex situ dataset). Locus statistics for each in situ population were calculated using the R package diveRsity v.1.9.90 (Keenan et al., 2013) and can be found in Table S4. Clones were identified with the R package poppr v.2.8.3 (Kamvar et al., 2014), and only one of each clone group was included in the further analysis; we do not investigate the influence of clones in this manuscript.

### 2.5 Analytical methods

#### 2.5.1 Aim 1: What is the percent of genetic diversity conserved ex situ, and what is the minimum sampling recommended?

This is a two part question and thus involves two approaches. First, we compare the genetic diversity from the actual ex situ dataset to the in situ dataset −we calculate the percent of wild alleles currently conserved ex situ. We perform a simulated subsampling, over all possible sample sizes, of the in situ dataset to represent an idealized sampling of wild populations by a collector who takes one seed or cutting per wild individual. This idealized sampling is used to calculate the minimum size needed to reach a threshold of 95% of the alleles (see also Hoban, 2019; Hoban & Strand, 2015). All analyses were performed in R v.3.6.3 (R Core Team, 2020). First, binned allele calls from Geneious were manually converted to a genepop file format. Genepop files were used to create ‘genind’ and ‘genpop’ objects in R with the package adegenet v.2.1.2 (Jombart, 2008). Using custom scripts (see Hoban, Callicrate, et al., 2020, for details; https://github.com/smhoban/IMLS_Safeguarding), we calculated the percentage of in situ alleles that were present in the ex situ gardens by pooling all individuals held at gardens. We calculated this separately for alleles in categories based on their frequencies as follows: ‘very common’ (>10%), ‘common’ (5%–10%), ‘low frequency’ (1%–5%), and ‘rare alleles’ (<1%), as well as ‘all alleles’. We focused on alleles as the measure of genetic conservation (as opposed to heterozygosity, for example) because they are the aspect of genetic variation on which natural selection can act (Brown & Hardner, 2000).

We also assessed alleles conserved for the East and West regions separately (see also Zumwalde et al., 2021) to determine whether genetic diversity is better conserved from one region or the other. We calculated how many “West alleles” (alleles present in West populations, e.g., all alleles minus alleles private to the East) were captured in ex situ seedlings taken from the West, and how many “East alleles” were captured in ex situ seedlings taken from the East. We used a Chi‐Square Test in R to determine if there was a significantly higher capture of alleles in the East compared to the West.

Secondly, we use simulated subsampling of wild populations. We used the optimization approach of Hoban and Schlarbaum (2014) to determine the minimum number of sampled individuals to achieve a given threshold of genetic diversity. This approach involved simulated subsampling of the entire in situ dataset for all possible sample sizes ranging from 1 to 667. This simulates collecting seeds from the wild in which a seed sampler selects plants randomly and takes one seed or cutting per plant; in contrast to the real ex situ dataset, this simulated ex situ collection will not have half‐sibling families. In other words, the minimum sample size is truly the minimum and is based on ideal sampling. For each subsample, the percentage of alleles captured in the subsample was calculated. The first subsample to exceed 95% (averaged over 75,000 replicates) of the in situ alleles was recorded as the minimum necessary sample size. Similar to previous works (Hoban, 2019; Hoban et al., 2018), we made this calculation using two different assumptions: that ‘all alleles’ in the in situ dataset are considered (i.e., full dataset) and that alleles present in two or fewer copies are dropped (i.e., reduced dataset). The reduced dataset essentially filters ultra‐rare alleles (i.e., those that occur only once or twice in the dataset), which may be deleterious or a potential result of genotyping errors, while the full dataset assumes all alleles have potential value (see Discussion in Hoban, Callicrate, et al., 2020).

#### 2.5.2 Aim 2: Comparison of genetic diversity conserved, and minimum sampling recommended between *Q. havardii* and 11 rare species

We used an allele accumulation curve (i.e., the percentage of alleles captured for each sample size) to compare *Q*. *havardii* to 11 other taxa from a recent study (Hoban, Callicrate, et al., 2020): two palms, two cycads, three oaks, two magnolias, and two hibiscuses. These taxa have a smaller range size and numeric census size than *Q*. *havardii*, with most having at maximum a few thousand known plants in situ. We overlaid on this a logarithmic regression (using the “lm” and “predict” functions in R) between the number of individuals currently held ex situ and the percentage of alleles they conserve, established in this prior study of rare species. If *Q*. *havardii* is below the relationship for these 11 rare species, it indicates that less genetic diversity is captured than expected from the number of individuals. The minimum sample size for *Q*. *havardii* was also compared to the minimum sample size for the 11 rare species, for which the same resampling procedure was applied.

#### 2.5.3 Aim 3: Genetic diversity and structure among botanic garden populations and relationship to the number of individuals

We then calculated the percentage of all in situ alleles present in each botanic garden. We used linear regression models in R to test for a relationship between the number of plants in a garden and the percentage of each category of allele captured. An examination of the residuals suggested that residuals of the linear model are not normally distributed for ‘all alleles’ and ‘low‐frequency alleles’, so we also tested for a relationship between log and square root transformations of the number of plants and the percentage of each category of the allele. Finally, we tested for a relationship between the number of accessions (i.e., maternal families consisting of sets of seeds from the same plant) and the percentage of each category of allele, and the number of populations sourced and each category of the allele. We acknowledge that each garden has various amounts of material from different regions, populations, and maternal lines, which may violate the assumption of linear regression (independence of samples).

We also calculated F_ST_ using the R package hierfstat v.0.4.22 (Goudet, 2005) between each garden and the East and the West regions. Then, to visualize garden populations in relation to in situ regions, we performed a Discriminant Analysis of Principal Components (DAPC) using the “dapc” function in the R package adegenet (Jombart et al., 2010). DAPC is a multivariate method for identifying and visualizing genetic clusters and the relationships between them (Miller et al., 2020).

#### 2.5.4 Aim 4: Calculation of geographic and ecological diversity conserved

We build on geographic methods introduced in Beckman et al. (2019, 2021) and Khoury et al. (2019; and previously in Khoury et al., 2015) for estimating the percentage of a species’ native range that is represented in ex situ collections. To make this calculation, we compared two sets of geographic points: all known in situ occurrences and ex situ collection source localities (e.g., wild occurrences where seeds were collected). The set of in situ occurrences was created from several large biodiversity databases including GBIF (gbif.org), FIA (fia.fs.fed.us), iDigBio (idigbio.org), and SEINET (swbiodiversity.org/). The set of ex situ occurrences was the set of localities where seeds were collected for ex situ conservation. We placed a circular buffer around each in situ occurrence point and each ex situ occurrence point. The buffer does not have a precise biological meaning but is meant to approximate potentially suitable habitats or nearby populations. Some authors use ecological niche models to produce a probabilistic projection of in situ suitable habitat (Khoury et al., 2020), but we choose not to because niche models are generally used to predict potentially suitable habitat and/or distribution and are not always a direct reflection of species’ occurrence. The area where ex situ buffers overlap with in situ buffers is considered ‘conserved’ in ex situ collections. We divide the area covered by buffers surrounding ex situ points by the total area covered by buffers surrounding in situ points and multiply by 100, resulting in a percentage of the geographic range that is conserved ex situ. We test three buffer sizes (10 km, 50 km and 100 km radii) to determine if our conclusions are affected by buffer size. Previous work has primarily used 50 km radius buffers (Khoury et al., 2020; Ramírez‐Villegas et al., 2010).

Next, we estimated ecological coverage by calculating the number of EPA Level III and IV Ecoregions (downloaded from https://www.epa.gov/eco‐research/ecoregions; Figure 2) that in situ *Q*. *havardii* populations (e.g., buffers) overlap with, and the number of ecoregions that ex situ samples are taken from (for a graphical representation, see Figures S3 and S4). EPA ecoregions are a synthetic concept of habitat that includes climate, soils, vegetation cover, hydrology, and geology (Omernik, 2004). Level IV ecoregions are the finest scale designation consisting of 967 total ecoregions in the continental United States. Conserving populations from each ecoregion is presumed to conserve local adaptations (Di Santo & Hamilton, 2020; Hanson et al., 2017). Both the geographic and ecological calculations were also made separately for the eastern and western populations. All calculations were performed in R (see Data Accessibility Statement). As a complementary approach to visualize how well ex situ samples cover the ecological variability of the species’ range, we used a Principal Component Analysis (PCA) of 13 uncorrelated environmental variables for in situ and ex situ occurrence points. Codes for the final uncorrelated variables used are as follows: BIO2 = mean diurnal range, BIO3 = isothermality, BIO4 = temperature seasonality, BIO5 = maximum temperature of the warmest month, BIO8 = mean temperature of wettest quarter, BIO10 = mean temperature of warmest quarter, BIO11 = mean temperature of coldest quarter, BIO12 = annual precipitation, BIO14 = precipitation of driest month, BIO15 = precipitation seasonality, BIO18 = precipitation of warmest quarter, and BIO19 = precipitation of coldest quarter.

## 3 RESULTS

Our microsatellite dataset had 2.6% missing data (Figure S5), and a total of 244 alleles were observed in situ and 186 alleles ex situ. Detailed genetic summary statistics for each in situ population can be found in Zumwalde et al. (2021), who showed that patterns of differentiation from genetic, morphological, and environmental datasets primarily corresponded to the disjunction of populations from the eastern and western regions of the species’ geographic range. Additionally, Zumwalde et al. (2021) noted that western populations generally had higher levels of genetic diversity and lower relatedness when compared to eastern populations.

### 3.1 Aim 1: How much genetic diversity is conserved ex situ, and what is the minimum sampling recommended?

For the reduced dataset (in which singletons and doubletons are dropped), we found that 79% of the overall species’ alleles are conserved in the 290 ex situ seedlings, with 100% of ‘very common’ and ‘common alleles,’ 94% of ‘low‐frequency alleles’, and 55% of ‘rare alleles’ captured (Table 1). There was a significant difference between conservation of the East and West regions (*p *= 0.032); 68% of the alleles from the West were captured compared to 93% of the eastern region. The results for the full dataset are similar though all values are lower (Table 1).

**TABLE 1:** Percentage of genetic diversity conserved ex situ, for each of five categories of alleles, for the East and West regions and overall values, using the reduced dataset (percent using the full dataset shown in parentheses)

	Number of samples	All alleles	Very common (>10%)	Common (5%−10%)	Low frequency (5%−1%)	Rare (>1%)
East	237	93% (82%)	100%	97%	98%	63% (61%)
West	53	68% (54%)	100%	98%	77%	30% (20%)
Overall	290	79% (70%)	100%	100%	94%	55% (48%)

Using simulated subsampling of the wild individuals, for the ‘reduced’ dataset, we found that the number of sampled individuals needed to reach a minimum of 95% of the alleles in the ‘all alleles’ category for *Q*. *havardii* is 245 (assuming sampling occurs in East and West from all populations). Considering the ‘reduced’ dataset, if a sampler only wanted to focus on the two regions separately, the minimum sample size for the East alleles is 101, while the minimum sample size for the West is 148. Meanwhile, under the conservative assumption of the full dataset, 481 sampled individuals would be needed overall, 217 for the East, and 284 for the West.

### 3.2 Aim 2: Comparison of genetic diversity conserved, and minimum sampling recommended between *Q. havardii* and 11 rare species

**FIGURE 3:**
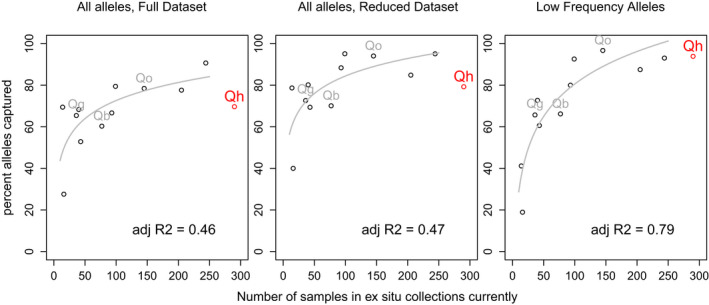
Genetic diversity captured (y‐axis) per number of individual plants ex situ (x‐axis) for the threatened but widespread *Quercus havardii* (red dot) and 11 previously investigated rare plant taxa (black open circles). Among these 11, three rare *Quercus* species are indicated with their initials in grey (*Q. georgiana*,*Q. oglethorpensis*, and *Q. boyntonii*). The grey line indicates a logarithmic regression on the 11 rare taxa previously established in Hoban et al. (2020)

**FIGURE 4:**
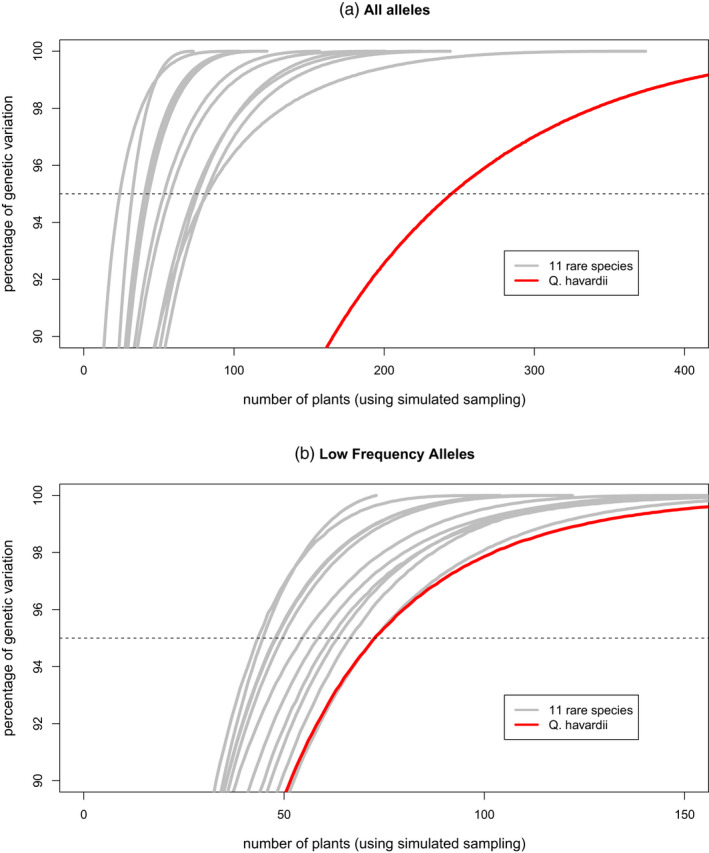
Genetic diversity expected to be captured (y‐axis) for a given simulated sampling intensity (x‐axis) for 11 previously studied rare species (grey lines) and *Quercus havardii* (red line) for two categories of alleles, using the reduced dataset (singleton and doubletons excluded): (a) all alleles and (b) low frequency alleles. Note that simulated sampling assumes ideal sampling conditions and visiting all populations and thus represents minimum sampling, and in the 'real world' sampling efforts would be substantially higher

A lower percent of the genetic diversity of *Q*. *havardii* was shown to be conserved compared to an expected allele accumulation curve for 11 rare, long‐lived species, including three rare *Quercus* species (Figure 3). Notably, it is below the percent conserved for *Quercus oglethorpensis*, despite having twice the number of plants ex situ. Using simulated sampling and the ‘reduced’ dataset, we found that the number of samples needed to reach a minimum of 95% of the alleles in the ‘all alleles’ category was much greater for *Q*. *havardii* (245) than for 11 rare species (mean of 56). The number of samples needed for *Q*. *havardii* for ‘low‐frequency’ alleles was also greater− 73 compared to a mean of 56 for the other 11 species (Figure 4).

### 3.3 Aim 3: Genetic diversity and structure among botanic garden populations and relationship to the number of individuals

**FIGURE 5:**
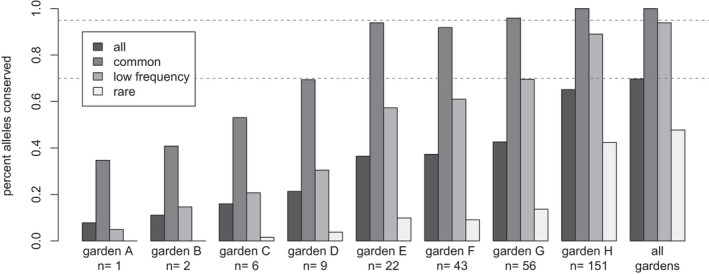
Percent of alleles conserved in different botanic gardens for three categories of alleles (see legend) using the reduced dataset (singleton and doubletons excluded). Thresholds of 70% and 95% alleles are indicated by dashed grey horizontal lines. The participating gardens are listed in Table S1

The percent of alleles captured in each of the eight botanic gardens is shown in Figure 5. There was a clear increase in genetic diversity captured with the number of individuals per garden. For the ‘common’ and ‘very common alleles’, there is a plateau at around 22 plants, which captured >90% of the genetic diversity. However, for the categories ‘all alleles’, ‘low‐frequency’ alleles, and ‘rare alleles’, genetic diversity continues to accumulate with more samples.

**FIGURE 6:**
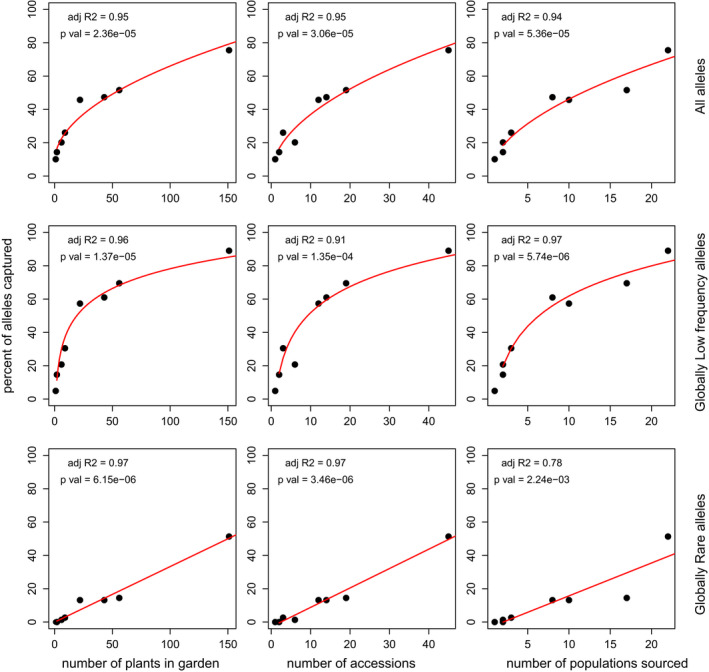
Percent of alleles (y‐axis) captured in a given population size (number of plants; x‐axis) of *Quercus havardii* currently held in botanic gardens for the reduced dataset (singletons and doubletons removed). For each plot a regression was performed using no transformation, square root transformation, and log transformation of number of plants, with the regression line shown and the adjusted R^2^ shown in the top left of each plot

There was a strong relationship between the number of plants in a garden and the percent of genetic diversity (Figure 6, Figures S8 and S9). Interestingly, a different data transformation of the number of plants (square root vs. linear vs. log) appears to be most appropriate (highest R^2^ value) for each kind of allele. The percentage of ‘all alleles’ conserved showed a strong relationship with the square root of the number of plants, low‐frequency alleles showed a log relationship, and rare alleles showed a linear relationship. There was also a strong relationship between the number of maternal families and the number of populations sampled (Figure 6).

On average, genetic differentiation between in situ and ex situ populations was significantly higher (paired *t*‐test; *p *= 0.049) when comparing each garden to the West region (mean pairwise *F*_ST_; *F*_ST_ = 0.0159) than when comparing each garden to the East (*F*_ST_ = 0.0066). Examination of BIC (Bayesian Information Criterion; Figure S6) values for *k* number of clusters did not reveal a clear single minimum, though *k* values between 25 and 40 were all quite low. This lack of a clear signal for a single *k* suggests a hierarchical structure as might be expected for a widespread species. We, therefore, do not conclude there is a “best” value of *k* for this dataset, but we set *k* = 2 as it is biologically plausible (see also Zumwalde et al., 2021) and will allow comparison of the garden and regional in situ samples. This differentiation was also visible in a DAPC plot (Figure S7) suggesting that the genetic composition of garden populations is more similar to eastern populations.

### 3.4 Aim 4: Calculation of geographic and ecological diversity conserved

Percentages reflecting conserved geographic area and ecoregion coverage are shown in Table 2. Generally, these percentages were lower than the estimates of genetic diversity conserved. The buffer size does impact the percentage considered conserved with smaller buffer sizes resulting in a lower percentage. Depending on the region and buffer size, geographic area percentage ranged from 11.32% to 42.29%, while ecological coverage percentages ranged from 50% to 90.91% for Ecological Level III, and 29.03% to 54.76% for Ecological, Level IV (Table 2). In almost all cases, the percentages conserved were higher in the West than in the East (the one exception being Ecological, Level III, 10 km buffer, Table 2). This trend is the opposite pattern found in the genetic diversity percentages. A PCA of environmental variables for the sampled sites visualizes this ecological coverage in two dimensions (Figure S10).

**TABLE 2:** Percentage of geographic area (km^2^) and ecological coverage (number of ecoregions) of ex situ samples for *Quercus havardii*, using three different methods (Geographic, and Ecological ecoregion levels III and IV), across three ranges (Overall [entire species distribution], East region only, and West region only), considering three buffer sizes (10 km, 50 km, and 100 km radii)

Method	Buffer (km)	Overall	East	West
Geographic	100	37.89% (303,123/800,037)	35.42% (181,768/513,111)	42.29% (121,354/286,926)
	50	24.25% (109,255/450,552)	20.90% (68,002/325,317)	32.94% (41,254/125,235)
	10	13.20% (7,269/55,074)	11.32% (5,070/44,794)	21.39% (2,199/10,280)
Ecological, Level III	100	84.62% (11/13)	62.50% (5/8)	66.67% (6/9)
	50	90.91% (10/11)	71.43% (5/7)	83.33% (5/6)
	10	63.64% (7/11)	66.67% (4/6)	50.00% (3/6)
Ecological, Level IV	100	50.45% (56/111)	37.04% (20/54)	52.94% (36/68)
	50	45.12% (37/82)	33.33% (14/42)	54.76% (23/42)
	10	35.29% (18/51)	29.03% (9/31)	42.86% (9/21)

Note

Values in parentheses for the geographic method indicate area conserved/total area, while for the ecological method they indicate the number of ecoregions conserved/total ecoregions, which are used to calculate the percentages.

## 4 DISCUSSION

Our results provide one of the first studies to quantify genetic conservation value of ex situ plants for a widespread but threatened species, examine genetic diversity and structure among botanic garden populations, and directly compare genetic and ecogeographic conservation. There is a growing need and opportunity for this research due to increasing environmental change and biodiversity loss, heightened conservation mission of botanic gardens (Cavender et al., 2015; Westwood et al., 2021), and increasing affordability and precision of genetic and geospatial techniques (Hoban et al., 2021; Paz‐Vinas et al., 2021). For ex situ collections to be of high conservation value, plant collection strategies need to ensure that the material sufficiently represents the diversity of the source populations (Brown & Hardner, 2000; Guerrant et al., 2014; Maunder et al., 2004). We found that (1) a majority of *Q*. *havardii* genetic diversity is conserved, though the genetic diversity of the eastern region is better conserved in ex situ botanic garden collections than of the western region; (2) genetic diversity conservation of this widespread species is lower than for 11 previously studied rarer taxa; (3) genetic diversity within each garden is strongly related to the number of plants in the garden; and (4) the measures of geographic and ecological conservation (i.e., percent area and percent of ecoregions represented in seed collections) were lower than the direct assessment of genetic diversity conservation (i.e., percent alleles).

Our first main conclusion is that the majority of *Q*. *havardii* genetic diversity, at least using microsatellite alleles, is conserved (Table 1). Also, our results suggest that successful genetic conservation will require more ex situ individuals from species with large geographic ranges than from rare species (Figures 2 and 3). The 290 ex situ plants in this study capture a lower percentage of genetic diversity than would be predicted from a relationship based on 11 previously studied rare species (including 3 rare oaks). As one example, *Q*. *havardii* has a lower percentage of alleles conserved (79%) than the rare oak *Q*. *oglethorpensis* (94%), which has an estimated extent of occurrence of 130,000 km^2^ compared to 300,000 km^2^ estimated for *Q. havardii* (Kenny et al., 2020 *)*, though *Q*. *oglethorpensis* has half as many trees (145) ex situ. However, both species possess similar values for the conservation of ‘low‐frequency alleles’ (94% and 97% respectively). The low percentage conserved for *Q*. *havardii*, therefore, relates to the fact that the number of rare alleles will be higher when the range size and census size are larger, as noted in Hoban (2019) and by others (Brown & Hardner, 2000; Brown & Marshall, 1995).

One reason for the relatively low amount of genetic diversity conserved is that *Q*. *havardii* is a widespread species with numerous populations. Hoban (2019) used simulations to demonstrate that more samples are needed when a species has more populations, larger populations, and a larger geographic range (and lower migration rates). Another reason relates to lower genetic conservation in the West. Gardens conserved fewer West alleles and had a greater genetic distance to western populations. During the initial seed collection, fewer populations were producing seed in the West, where average rainfall is much lower (less than half than annual rainfall as well as total precipitation in the wettest season, see Zumwalde et al., 2021). A total of 194 seeds were collected in the West, of which 53 seedlings were produced and analyzed, while 1557 seeds were collected in the East, of which 237 seedlings were produced. In addition, resampling analysis revealed that more sampled individuals are needed for conserving Western genetic diversity (for East it is 217, and West it is 284, for the ‘full’ dataset, see Methods and Results). For future collections, a larger number of samples from the West would improve the total genetic diversity captured.

Genetic diversity was strongly predicted by the number of individual plants in a garden, as well as the number of maternal accessions and by the number of populations, though the form of the relationship depended on the category of the allele. This was likely because the seed was distributed to botanical gardens from numerous populations and as many maternal plants as possible. Interestingly, garden E has more alleles than garden F (Figure 5, see also Tables S5 and S6), though only about half the number of individuals. Garden E has seeds from 10 populations (6 East, 4 West), while garden F has seeds from only 8 populations (7 East, 1 West), emphasizing that sampling from as many populations and regions as possible is important. We also observe that even small collections (e.g., 20 trees) have value for conserving ‘common alleles,’ and the total collection of all gardens together (i.e., the metacollection) contains more genetic diversity than any individual population (as suggested by Griffith et al., 2019).

Previous studies have recommended a range of minimum number of samples for conserving genetic diversity. While investigating *Zamia lucayana* and *Z*. *decumbens* (Zamiaceae), Griffith et al. (2017) found that a single accession (a group of seeds from one maternal plant) contained 24%–51% of the alleles, while the total collection captured 90%. For *Leucothrinax morrisii* (Arecaceae), Namoff et al. (2010) showed that a collection of as few as 15 ex situ plants was required to reach 80% allelic capture, while 58 plants captured 93%. McGlaughlin et al. (2015) recommended 60 or 125 individuals of *Sibara filifolia* (Brassicaceae) to capture 90% of ‘all alleles,’ depending on the target population. This literature, combined with our results herein and our previous studies (Hoban, 2019; Hoban, Callicrate, et al., 2020), show that there is not a single appropriate collection size, and the number of sampled individuals needed depends on the species’ biology as well as the spatial scope (e.g., a focal population, region, or the entire range), the type of allele, and assumptions such as minor allele threshold. A minimum of 100–200 individual plants may suffice for rare species, but more are needed for widespread threatened species. We note that guidance on minimum sample size should include not only a minimum number of sampled individuals but also a minimum number of populations or portion of the species range, a topic that future investigations should explore− here as with much previous work we assume a collector can reach all or most populations. The best practice remains to collect seeds from more than 25–50 individuals per population (when possible) and that sampled individuals are well‐dispersed throughout the population (to avoid close relatives and the impact of fine‐scale spatial genetic structure).

This guidance regarding minimum sample size and representation across populations is especially vital for species that produce recalcitrant seeds (which do not tolerate desiccation for storage in conventional seed banks) because they must be held in living collections, requiring significant space. For species that require hundreds of individuals to sufficiently capture wild genetic diversity, and especially for larger specimens such as shrubs or trees, coordinated networks of living collections (metacollections) are required (Griffith et al., 2019). Botanic Gardens Conservation International has answered this challenge by creating Global Conservation Consortia– networks of many institutions working together to collectively conserve priority threatened plant groups (https://www.globalconservationconsortia.org). The Global Conservation Consortium for Oaks is one of eight current consortia, and its members work together to prioritize species, populations, and conservation actions (including for *Q*. *havardii*) while maintaining diverse living collections across tens of institutions.

Our study is one of the first comparisons of genetic and ecogeographic diversity conserved ex situ. We find that geographic and ecological measures of conservation success are typically much lower than genetic measures (e.g., the percentage of alleles conserved), with some exceptions for EPA Level III ecoregions. This is not entirely unexpected as other work has shown that populations can strongly decline in geographic extent or abundance (Alsos et al., 2012; Hoban et al., 2014) without substantial losses of genetic diversity. This is partly because genetic diversity is shared among populations through gene flow and shared ancestry, with especially high within‐population diversity in trees (Petit & Hampe, 2006). Additionally, although we have a large in situ sample, we did not collect from all populations nor comprehensively from most populations, and thus our count of alleles existing in situ, particularly rare alleles, may be incomplete. Thus, our estimates of the proportion of wild genetic diversity conserved for ‘all alleles’ are optimistic; if wild alleles exist that we have not observed, then the extent of allele conservation in current ex situ samples may be even lower. We also acknowledge that there may be occurrence records not known (given the species does exist in remote areas without roads or trails), and therefore we may underestimate the distribution and thus ecological and geographic diversity of the species.

Another explanation for lower percentages of geographic diversity conserved than genetic (allelic) diversity may be that geographic analysis counts a location as ‘conserved’ if *any* samples were taken from it while the genetic analysis better reflects if numerous samples were collected. The number of populations and number of seeds both affect genetic diversity captured. For instance, although a greater proportion of geographic area was covered in the West (Table 2), there were fewer seeds taken per population in the West. As such, genetic diversity conserved in the West is lower than that of the East, while geographic coverage in the West is higher. A future improvement to the ecogeographic approach would be to add weight to sites where more samples were taken. We cannot say which method is most appropriate to guide conservation genetic action (genetic, geographic, or ecological) as this will require further investigation and consideration of each species’ circumstances. We do suggest that the geographic method, being always lower than genetic diversity, is more conservative. From this logic, we speculate that the estimates of Khoury et al. (2019) that an average of only 3.3% of nearly 7000 crop wild relative species’ geographic ranges have been conserved effectively ex situ may not accurately depict, but rather underestimate, alleles captured ex situ.

### 4.1 Caveats and other remarks

An important caveat of this study is that eleven microsatellites are limited in their resolution; the oak genome likely contains 30,000 to 80,000 protein coding genes (Plomion et al., 2018; Ramos et al., 2018). Microsatellites are also typically non‐coding DNA (though see Lind‐Riehl et al., 2014), which may unlikely reflect adaptive genetic diversity. In addition, our estimates of genetic conservation are a snapshot in time, and they will change as seedlings in botanic gardens die and/or as new seed collections occur. Finally, our resampling technique chooses one sample (one seed or one cutting) per individual, while most seed samplers will realistically take multiple seeds per individual. As noted previously (Hoban, Callicrate, et al., 2020; Hoban et al., 2018), the minimum sample size to reach 95% of alleles is an absolute minimum, and seed samplers who sample multiple seeds per maternal plant should often aim to collect twice as much under realistic conditions (Hoban & Strand, 2015).

We note that the seedlings of *Q*. *havardii* in this study, like most ex situ collections, contain numerous half‐siblings and possibly fullsiblings. Previous work has suggested that relatedness will reduce genetic diversity conserved (Hoban & Schlarbaum, 2014), but the question of how the degree to which different sets of siblings (e.g. number of maternal families) will impact genetic conservation success requires further study, ideally using simulated data with many arrangements of family size.

Similar to previous work, we separate alleles into categories based on their frequencies. As expected, ‘all’ alleles, ‘low frequency’ alleles, and ‘rare’ alleles are harder to conserve compared to ‘very common’ alleles and ‘common’ alleles. It is not known if rare alleles are potentially advantageous, deleterious, or neutral. The precautionary principle in conservation would argue for the capture of rare alleles to maintain genetic diversity as a potential resource for nature and for people, especially as environmental pressures change, including threats of new pests and diseases. However, an opposing view is that ultra‐rare alleles may be deleterious and should not be preserved (Brown & Kelly, 2020; Kardos & Shafer, 2018), though neutral nuclear microsatellites are unlikely to be linked to deleterious alleles. Consensus on the importance of rare alleles is needed to determine practical guidelines for sampling. A valuable area of future work will be to apply the methods we used to a dataset containing alleles that are known or putatively under selection.

We also note that genetic diversity is not the only concern of a collection. Another important need is having enough plants to start a new population, based on expected germination and survival rates (Cochrane et al., 2007; Hoban & Way, 2016). As one example, to reach a restoration goal of 10 populations and mature plant numbers exceeding 1,000 per population would require almost 125,000 seeds of *Banksia ionthocarpa* subsp. *ionthocarpa* (Proteaceae; Cochrane et al., 2007). If *Q*. *havardii* were being collected for restoration, many thousands of seeds would be needed, as many seeds were desiccated or infested by pests, and numerous seedlings died. Furthermore, hybridization is common in oaks, so seedlings of *Q*. *havardii* should be checked phenotypically and genetically for any signs of introgression, in locations where other oaks were observed. Finally, to maintain the local adaptation of different populations, it may be advisable to plant individuals from different populations, regions, or environmental conditions in different sublocations in a botanic garden, or among botanic gardens.

### 4.2 Future directions

As next generation sequencing costs continue to decrease and oak genomic resources increase (Plomion & Martin, 2020), future studies have an opportunity to focus on coding regions of DNA that ultimately determine phenotypes of importance. Because nuclear microsatellites do not assess adaptive variation, a future study could include genes with possible physiological or morphological importance (e.g., water use efficiency, stomatal density, shape, and size of the leaves, etc.), or loci identified in gene−environment scans (Gugger et al., 2021), to analyze populations found in different ecoregions or areas of environmental space. This would allow comparison of genetic diversity captured in botanic gardens according to neutral genetic diversity, adaptive genetic diversity, and ecogeographic diversity. Another future direction is to establish common gardens of *Q*. *havardii* to test for local adaptation and predict response to climate change. It may be possible to identify which populations are more vulnerable or those unlikely to tolerate climate change and prioritize the samples for more seed collections (Borrell et al., 2020; Razgour et al., 2019).

While the botanic garden community is working together to conserve *Q*. *havardii*, there is still a need for further collections from the wild, particularly from western populations. In addition to increased sampling, Fant et al. (2016) and Wood et al. (2020) argue that arboreta and botanic gardens should take cues from the zoo community by improving shared databases of inventories for rare species, involving local communities in situ (or near in situ) to help conserve and sample the species, and sharing genetic material among gardens and herbaria for future studies.

Overall, our results show that higher sampling is needed in widespread species and that multiple types of data can reveal gaps in the ex situ collection (e.g., low genetic diversity from the West, low ecogeographic diversity from the East). It is worthwhile and important to continue collecting additional seeds from additional locations for a larger ex situ metacollection of this species (which could occur across multiple years due to relatively infrequent mast years of large seed production in oaks). The data collected on such future efforts, including sampling across multiple years, will inform future seedsampling expeditions to ensure that intraspecific diversity of threatened plants can be conserved.

## CONFLICT OF INTEREST

The authors declare no conflict of interest.

## Supporting information

Supplementary Material

## Data Availability

Data and code for the genetic analysis can be found here: https://github.com/smhoban/Qhavardii_ex_situ. Data and code for the environmental analysis can be found here: https://github.com/BZumwalde/Quercus_havardii_Safeguarding_genetic_diversity. Data and code for the geographic and ecological conservation analysis can be found here: https://github.com/esbeckman/Quercus_havardii_GeoEco_exsitu_conservation. All samples were collected and analyzed within the United States and the research described in the publication complies with relevant national laws implementing the Convention on Biological Diversity and Nagoya Protocol agreements. Benefits generated: During sample collection in situ we met with numerous local stakeholders and discussed the project. A report on the collection was distributed to all who participated. For sample collection ex situ we explained the project to the botanic gardens personnel involved. This report will be distributed to them upon completion. Their contributions are acknowledged. The research addresses a priority concern of many botanic gardens, especially those safeguarding this species, and a concern of the IUCN Red List, and clear conservation recommendations are made. Finally, as described above, all data and code have been shared with the broader public via appropriate biological databases for reproducibility.
