# Supplementary material for: Assessing ex situ genetic and ecogeographic conservation in a threatened but widespread oak after range‐wide collecting effort
Source: Evol Appl. 2022 May 31;15(6):1002–17. doi: 10.1111/eva.13391 (PMC9234636; doi:10.1111/eva.13391)
Supplement: Supplementary file 1 — Supplementary Material [file EVA-15-1002-s001.pdf]

Assessing ex situ genetic and ecogeographic conservation in a threatened but widespread oak after range-wide collecting effort

Table of Contents

**Fig. S1-** Seed collection histograms	**Page 2**
**Fig. S2-** Locus statistics: index of association	**Page 3**
**Fig. S3 and S4-** Ecoregions Level III, IV	**Page 4**
**Fig. S5-** Locus statistics: missing data	**Page 5**
**Fig. S6 and S7-** BIC for clusters, and DAPC	**Page 6**
**Fig. S8-** Percent alleles by plants, accessions	**Page 7**
**Fig. S9-** Percent alleles by plants, geographic region	**Page 8**
**Fig. S10-** Environmental PCA	**Page 9**
**Table S1-** Full list of individuals by garden	**Page 10**
**Table S2-** Primer information	**Page 18**
**Table S3-** Locus statistics: null allele checks	**Page 19**
**Table S4-** Locus statistics for each population	**Page 20**
**Table S5 and S6-** Results by garden	**Page 28**
**Methods S1-** Modified DNA extraction protocol	**Page 29**
**References**	**Page 31**

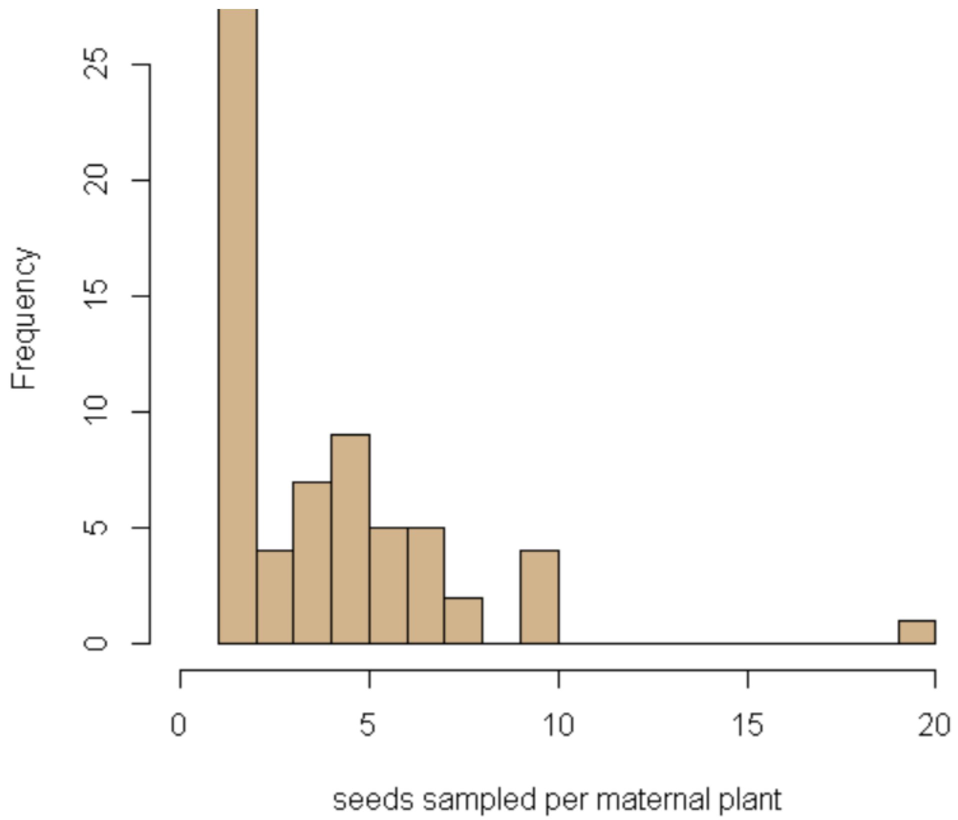

Figure S1. Histogram of number of seeds collected per maternal plant from the wild populations for the samples used in this study. Consistent with best practices from the Center for Plant Conservation and literature (e.g., Hoban & Schlarbaum, 2014), most plants had fewer than five seeds sampled, which better captures genetic diversity of the overall population. Two plants had >20 seeds collected.

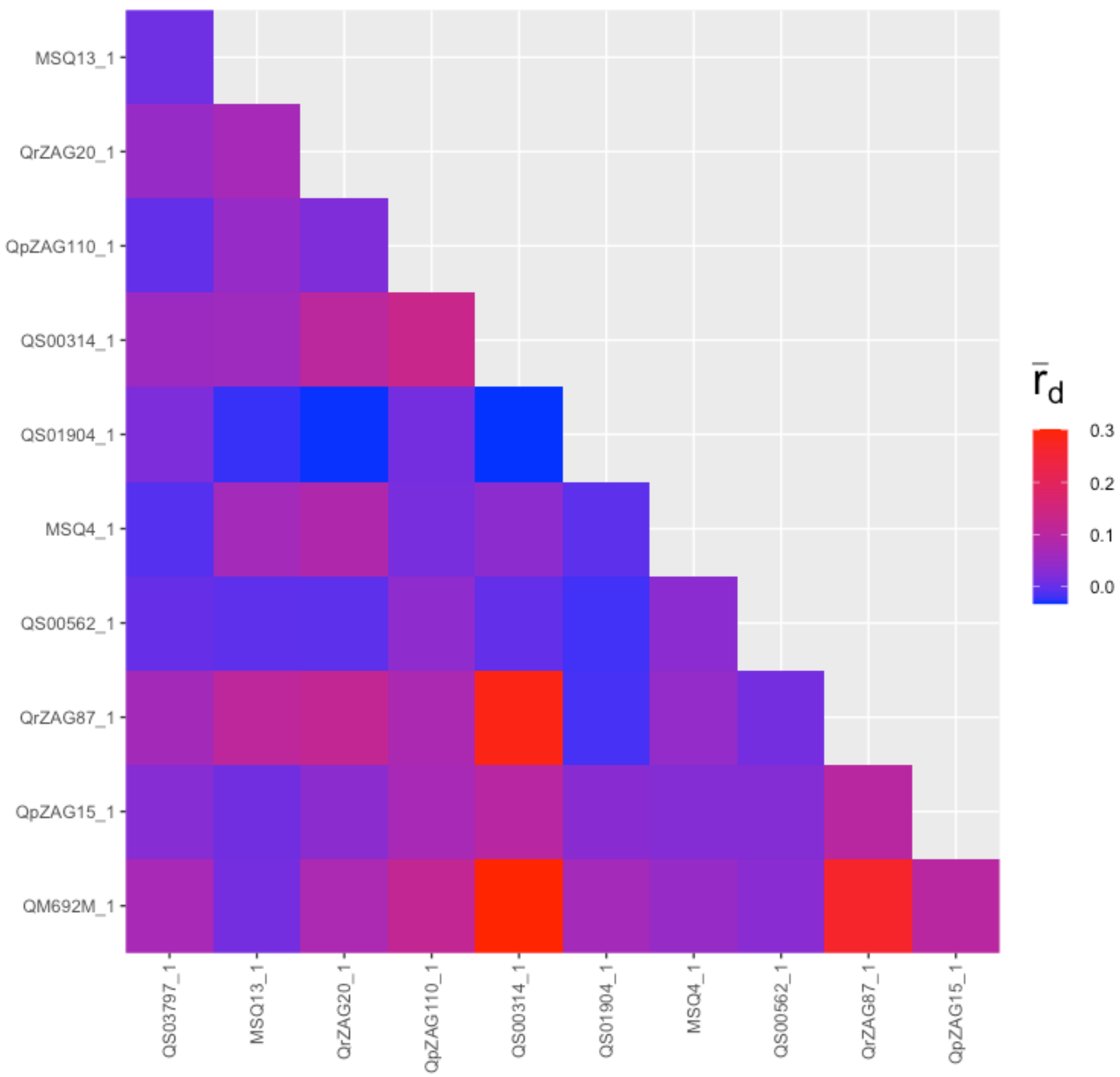

Figure S2. A heatmap showing the Agapow & Burt (2001) index of association, $\bar{r}_d$, for each pair of loci corresponding to the *Quercus havardii* in situ dataset.

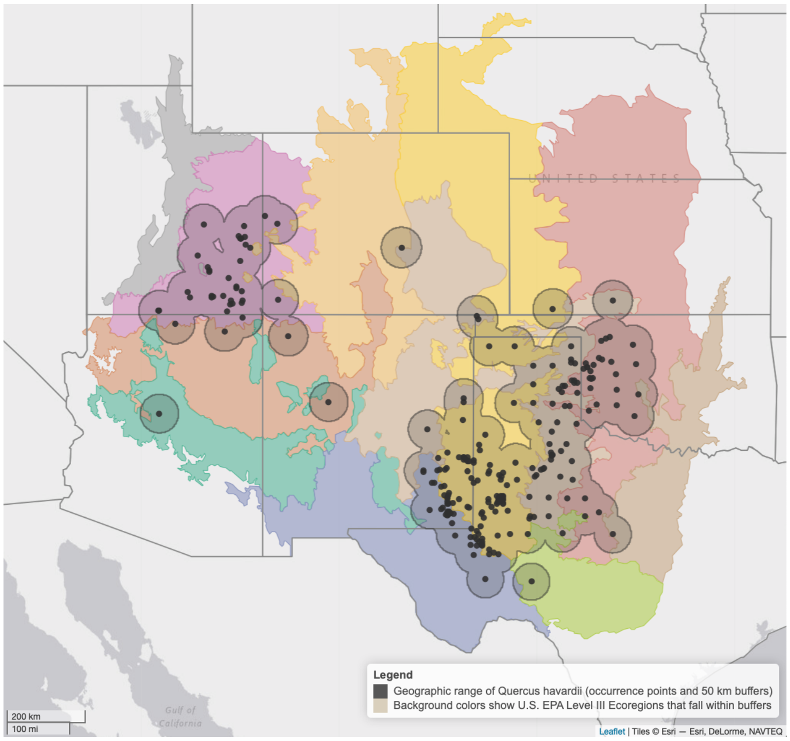

Figure S3. U.S. EPA Level III Ecoregions in which the species' range (as determined by a buffer surrounding the occurrence point) occurs.

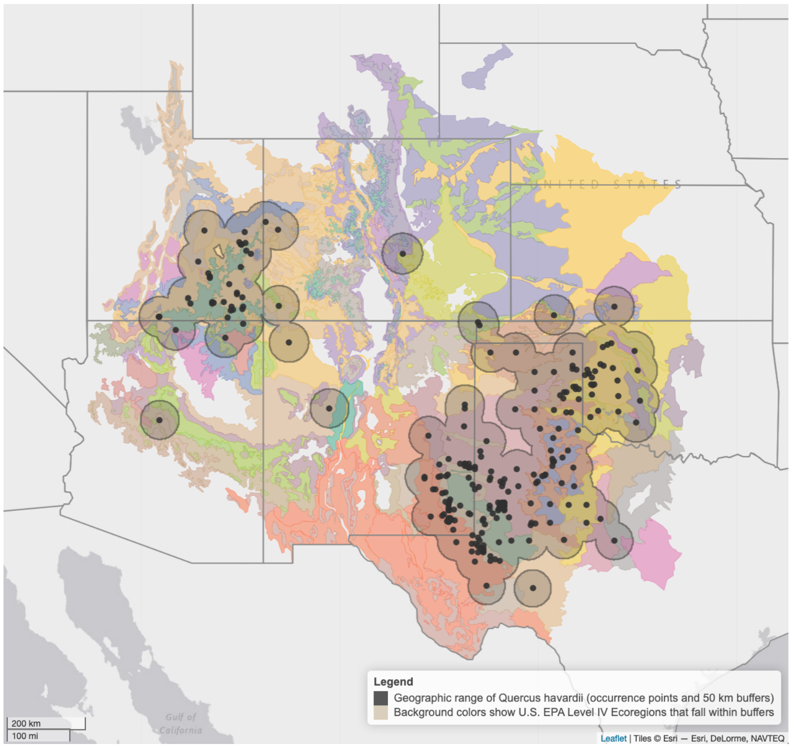

Figure S4. U.S. EPA Level IV Ecoregions in which the species' range (as determined by a buffer surrounding the occurrence point) occurs.

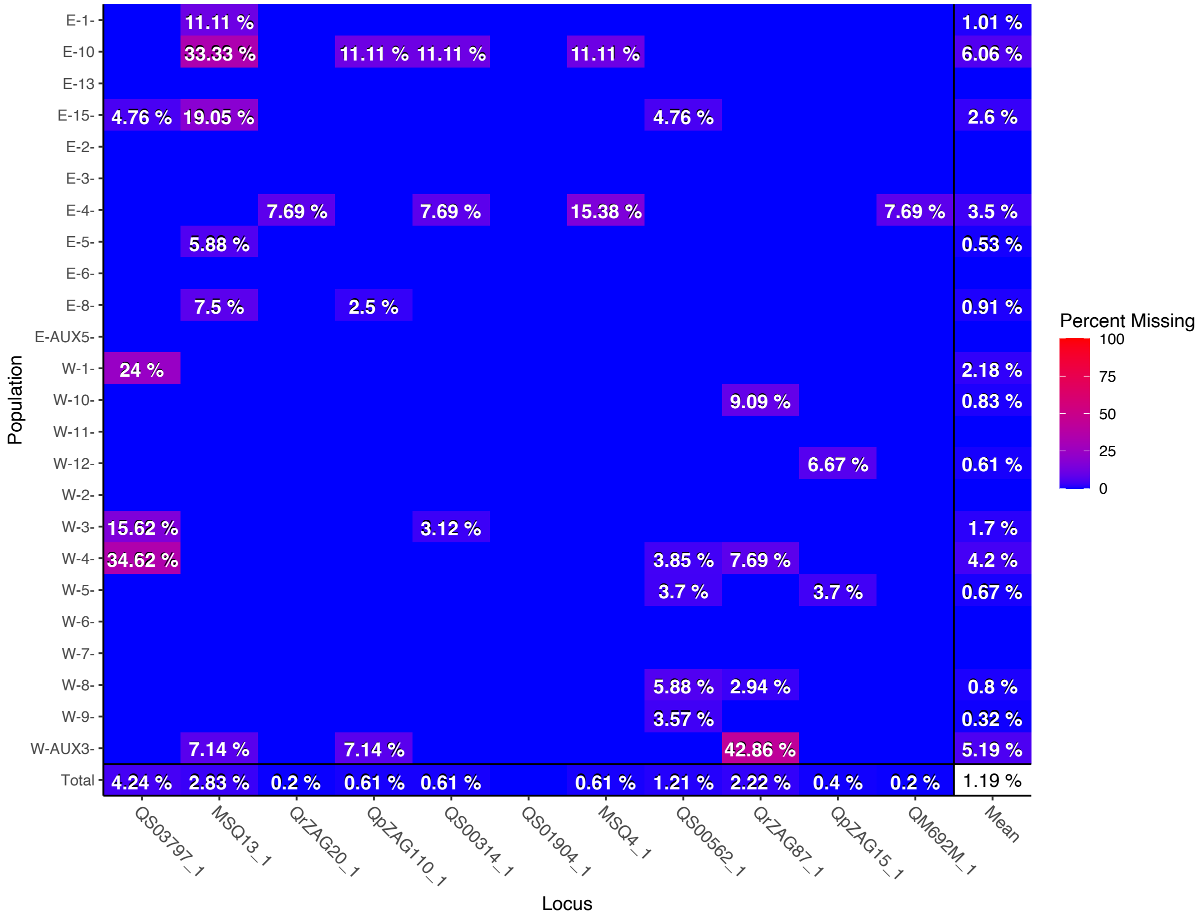

Figure S5. Locus summary for the percentage of missing data corresponding to the *Quercus havardii* in situ dataset.

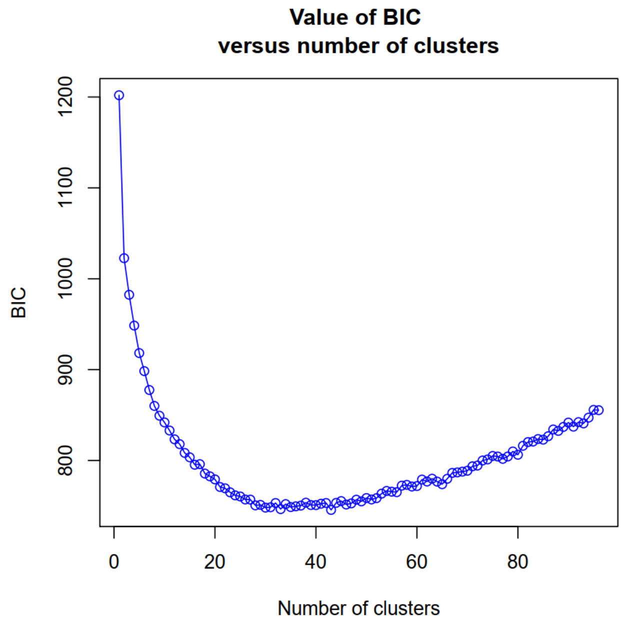

Figure S6. BIC and number of clusters evaluated for DAPC analysis.

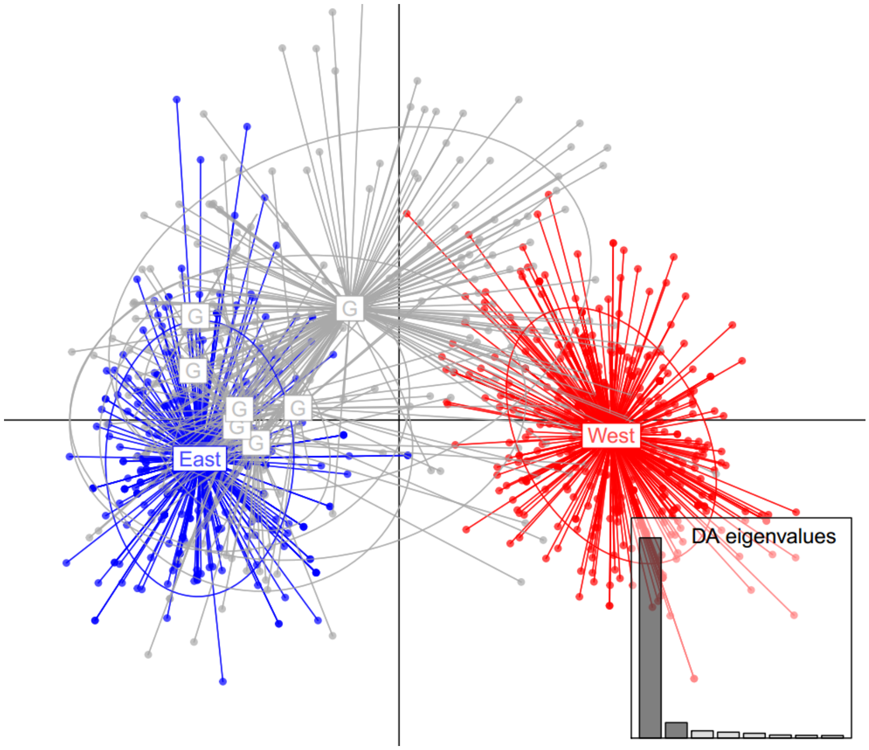

Figure S7. DAPC based on genetic data showing the location of each garden population (“G”; colored in grey) relative to the wild East (blue) and West (red) individuals.

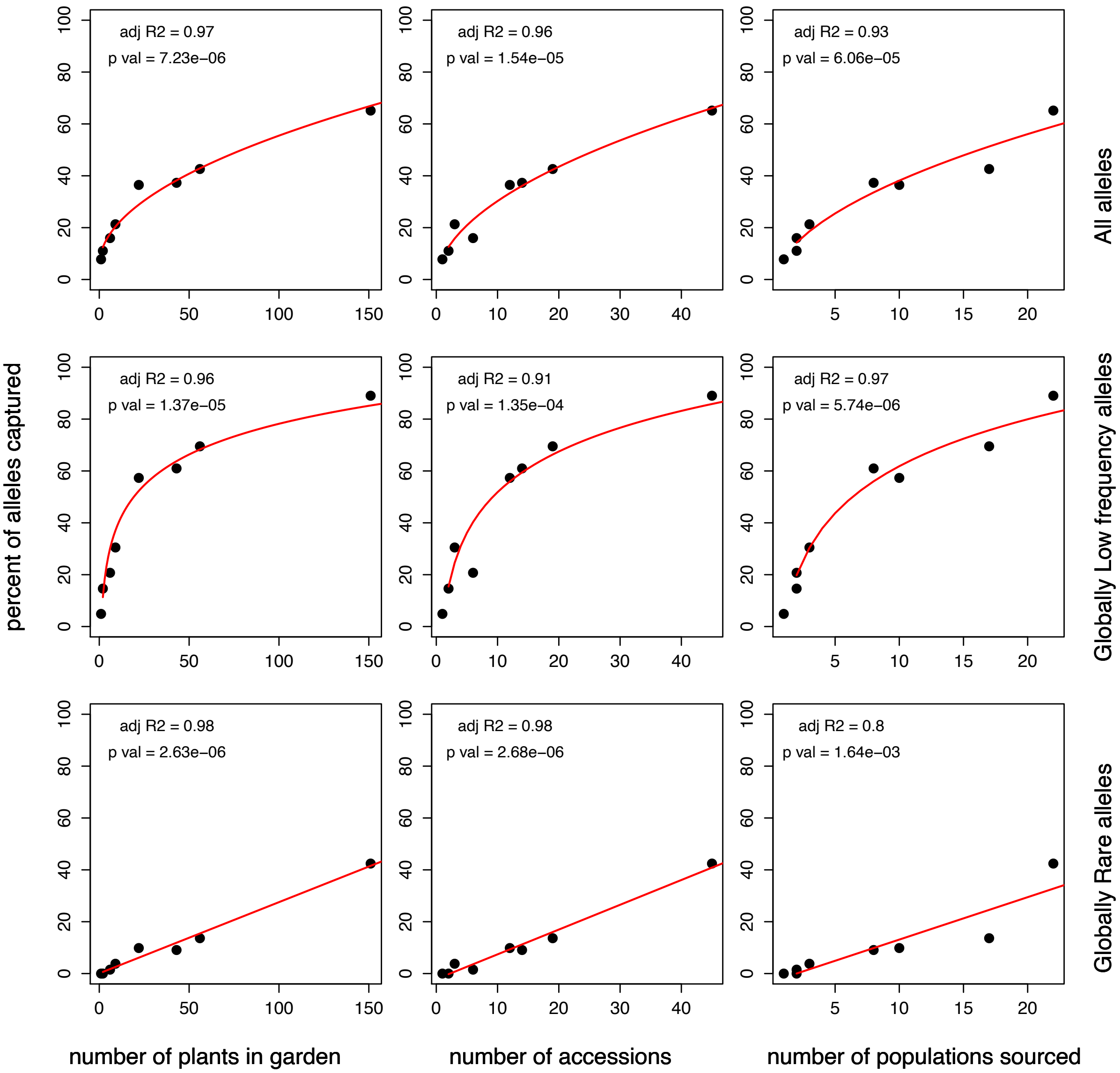

Figure S8. Percent of alleles (y-axis) captured for a given population size (number of plants, x-axis) of *Quercus havardii* currently held in botanic gardens, for the full dataset (singletons and doubletons included- complement to Figure 6). For each plot a regression was performed using no transformation, square root transformation and log transformation of number of plants, with the regression line shown and the adjusted R^2 shown in the top left corner of each plot.

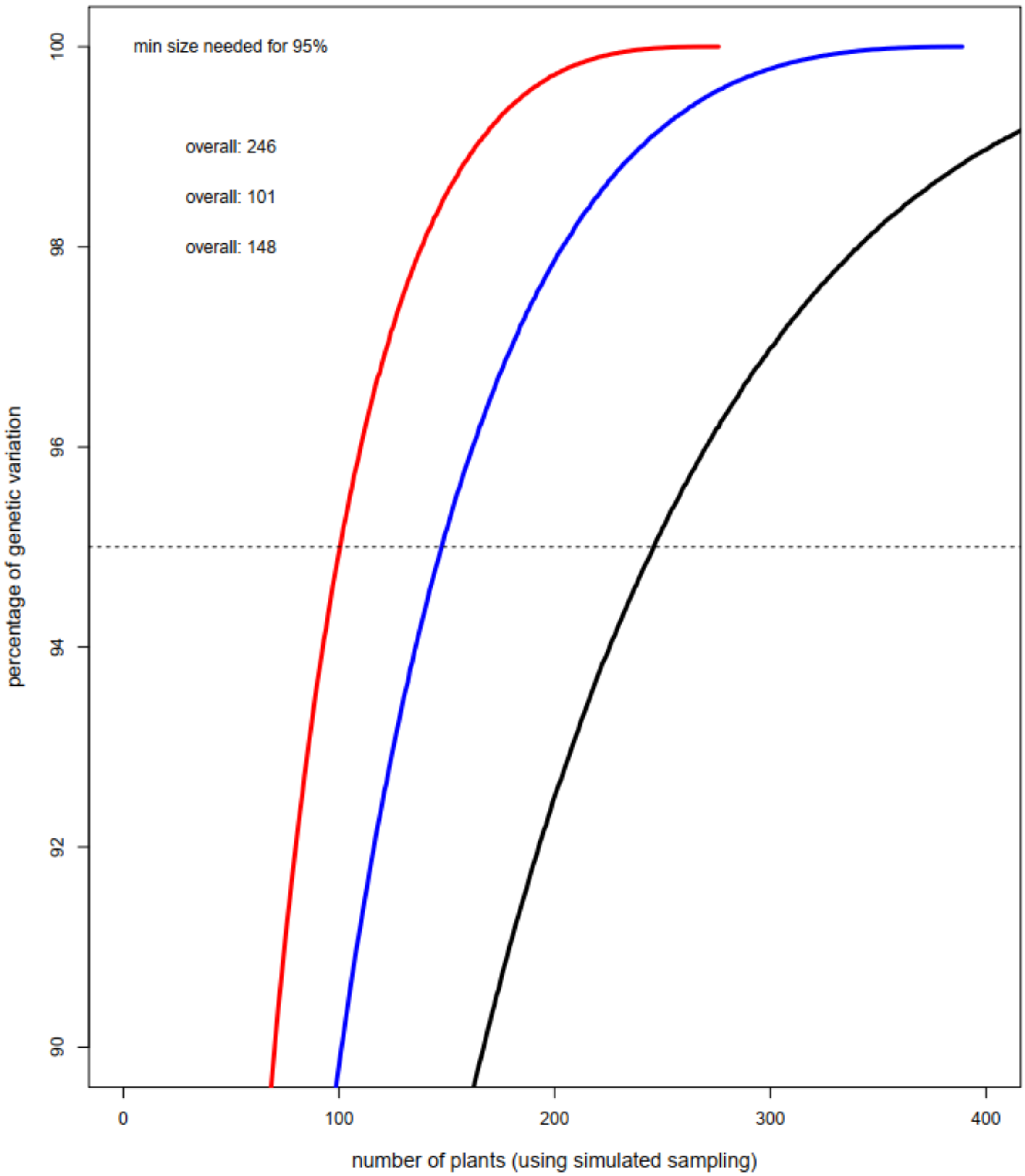

Figure S9. Percent of genetic variation (alleles; y-axis) captured for a given population size (number of plants, x-axis) for all samples across the geographic range (red line), samples from the East only (blue line), and samples from the West only (black line) of *Quercus havardii* currently held in botanic gardens, for the reduced dataset (singletons and doubletons removed- complement to Figures 6 and S8). Note, the minimum needed considering the 'rarest alleles dropped' overall is 246, while for East it is 101, and West it is 148.

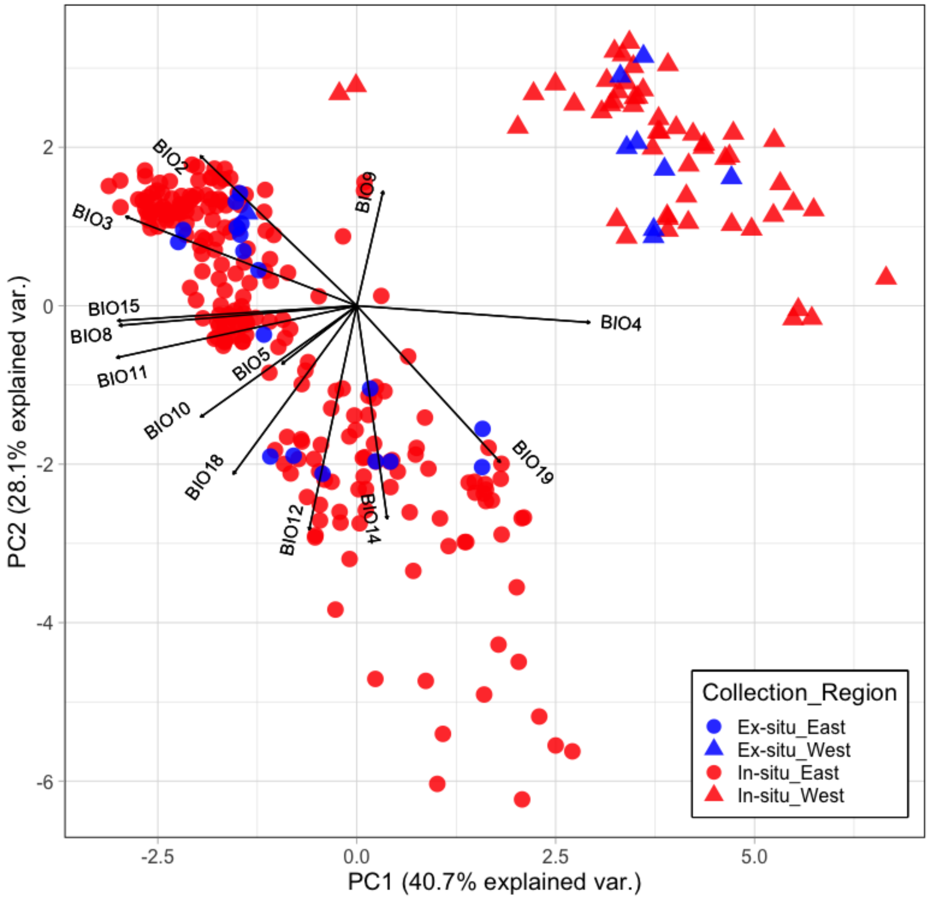

Figure S10. PCA based on 13 uncorrelated bioclimatic variables and populations *Quercus havardii*. Ex-situ populations (blue) and collected in-situ populations (red) represent sampling across its eastern (circles) and western (triangles) regions of the disjunct geographic range.

Table S1. A full list of all individuals found in each botanic garden, and their geographic source (region-population-accession).

DNA name	Tissue name	Accession Number	Region-Population-MaternalTree	Arboretum
SH-Q1392	QH-G0001	QH-E-5-1.1	QH-E-5-1	The Morton Arboretum
SH-Q1393	QH-G0002	QH-E-5-1.2	QH-E-5-1	The Morton Arboretum
SH-Q1394	QH-G0003	QH-E-5-9	QH-E-5-9	The Morton Arboretum
SH-Q1395	QH-G0004	QH-E-10-8.1	QH-E-10-8	The Morton Arboretum
SH-Q1396	QH-G0005	QH-E-11-1.1	QH-E-11-1	The Morton Arboretum
SH-Q1397	QH-G0006	QH-E-11-1.2	QH-E-11-1	The Morton Arboretum
SH-Q1398	QH-G0007	QH-E-11-1.3	QH-E-11-1	The Morton Arboretum
SH-Q1399	QH-G0008	QH-E-11-1.4	QH-E-11-1	The Morton Arboretum
SH-Q1400	QH-G0009	QH-E-11-4.2	QH-E-11-4	The Morton Arboretum
SH-Q1401	QH-G0010	QH-E-11-4.3	QH-E-11-4	The Morton Arboretum
SH-Q1402	QH-G0011	QH-E-11-4.10	QH-E-11-4	The Morton Arboretum
SH-Q1403	QH-G0012	QH-E-11-4.11	QH-E-11-4	The Morton Arboretum
SH-Q1404	QH-G0013	QH-W-AUX3	QH-W-AUX3	The Morton Arboretum
SH-Q1405	QH-G0014	QH-W-3-16	QH-W-3-16	The Morton Arboretum
SH-Q1406	QH-G0015	QH-W-3-14	QH-W-3-14	The Morton Arboretum
SH-Q1407	QH-G0016	QH-E-AUX6-1.2	QH-E-AUX6-1	The Morton Arboretum
SH-Q1408	QH-G0017	QH-W-3-1.4	QH-W-3-1	The Morton Arboretum
SH-Q1409	QH-G0018	QH-W-AUX10-2.7	QH-W-AUX10-2	The Morton Arboretum
SH-Q1410	QH-G0019	QH-W-AUX10-3.13	QH-W-AUX10-3	The Morton Arboretum
SH-Q1411	QH-G0020	QH-W-AUX10-3.14	QH-W-AUX10-3	The Morton Arboretum
SH-Q1412	QH-G0021	QH-W-AUX10-3.11	QH-W-AUX10-3	The Morton Arboretum
SH-Q1413	QH-G0022	QH-W-AUX10-3.12	QH-W-AUX10-3	The Morton Arboretum
SH-Q1414	QH-G0023	QH-W-3-1.6	QH-W-3-1	The Morton Arboretum
SH-Q1415	QH-G0024	QH-W-3-28.1	QH-W-3-28	The Morton Arboretum
SH-Q1416	QH-G0025	QH-W-6-30	QH-W-6-30	The Morton Arboretum
SH-Q1417	QH-G0026	QH-W-AUX10-3.20	QH-W-AUX10-3	The Morton Arboretum
SH-Q1418	QH-G0027	QH-W-3-17	QH-W-3-17	The Morton Arboretum
SH-Q1419	QH-G0028	QH-W-1-39	QH-W-1-39	The Morton Arboretum
SH-Q1420	QH-G0029	QH-E-15-1.1	QH-E-15-1	The Morton Arboretum
SH-Q1421	QH-G0030	QH-E-15-3.2	QH-E-15-3	The Morton Arboretum
SH-Q1422	QH-G0031	QH-E-15-5.2	QH-E-15-5	The Morton Arboretum
SH-Q1423	QH-G0032	QH-E-15-12.1	QH-E-15-12	The Morton Arboretum
SH-Q1424	QH-G0033	QH-E-15-10.2	QH-E-15-10	The Morton Arboretum
SH-Q1425	QH-G0034	QH-E-15-3.1	QH-E-15-3	The Morton Arboretum
SH-Q1426	QH-G0035	QH-E-15-9.1	QH-E-15-9	The Morton Arboretum

SH-Q1427	QH-G0036	QH-E-14-2.1	QH-E-14-2	The Morton Arboretum
SH-Q1428	QH-G0037	2015.004_1 Cannon	Other-source (not our collection)	The Morton Arboretum
SH-Q1429	QH-G0038	2015.003_1 Cannon	Other-source (not our collection)	The Morton Arboretum
SH-Q1430	QH-G0039	2015.001_1 Cannon	Other-source (not our collection)	The Morton Arboretum
SH-Q1123	QH-G0040	2015.003_2 Cannon	Other-source (not our collection)	The Morton Arboretum
SH-Q1124	QH-G0041	2015.003_3 Cannon	Other-source (not our collection)	The Morton Arboretum
SH-Q1125	QH-G0042	2015.003_4 Cannon	Other-source (not our collection)	The Morton Arboretum
SH-Q1126	QH-G0043	2015.004_2 Cannon	Other-source (not our collection)	The Morton Arboretum
SH-Q1127	QH-G0044	2015.003_5 Cannon	Other-source (not our collection)	The Morton Arboretum
SH-Q1128	QH-G0045	2015.001_2 Cannon	Other-source (not our collection)	The Morton Arboretum
SH-Q1129	QH-G0046	2015.001_3 Cannon	Other-source (not our collection)	The Morton Arboretum
SH-Q1130	QH-G0047	2015.001_4 Cannon	Other-source (not our collection)	The Morton Arboretum
SH-Q1131	QH-G0048	2015.003_6 Cannon	Other-source (not our collection)	The Morton Arboretum
SH-Q1132	QH-G0049	2015.001_5 Cannon	Other-source (not our collection)	The Morton Arboretum
SH-Q1133	QH-G0050	2015.001_6 Cannon	Other-source (not our collection)	The Morton Arboretum
SH-Q1134	QH-G0051	2015.001_7 Cannon	Other-source (not our collection)	The Morton Arboretum
SH-Q1135	QH-G0052	2015.001_8 Cannon	Other-source (not our collection)	The Morton Arboretum
SH-Q1136	QH-G0053	2015.001_9 Cannon	Other-source (not our collection)	The Morton Arboretum
SH-Q1137	QH-G0054	2015.001_10 Cannon	Other-source (not our collection)	The Morton Arboretum
SH-Q1138	QH-G0055	2015.001_11 Cannon	Other-source (not our collection)	The Morton Arboretum
SH-Q1139-2	QH-G0056	2015.001_12 Cannon	Other-source (not our collection)	The Morton Arboretum
SH-Q1140	QH-G0057	2015.001_13 Cannon	Other-source (not our collection)	The Morton Arboretum
SH-Q1141	QH-G0058	2015.0_1 Cannon	Other-source (not our collection)	The Morton Arboretum
SH-Q1142	QH-G0059	2015.001_14 Cannon	Other-source (not our collection)	The Morton Arboretum
SH-Q1143	QH-G0060	2015.001_15 Cannon	Other-source (not our collection)	The Morton Arboretum
SH-Q1144	QH-G0061	2015.001_16 Cannon	Other-source (not our collection)	The Morton Arboretum
SH-Q1145	QH-G0062	2015.001_17 Cannon	Other-source (not our collection)	The Morton Arboretum
SH-Q1146	QH-G0063	2015.001_18 Cannon	Other-source (not our collection)	The Morton Arboretum
SH-Q1147	QH-G0064	2015.001_19 Cannon	Other-source (not our collection)	The Morton Arboretum
SH-Q1148	QH-G0065	2015.003_7 Cannon	Other-source (not our collection)	The Morton Arboretum
SH-Q1149	QH-G0066	2015.003_8 Cannon	Other-source (not our collection)	The Morton Arboretum
SH-Q1150	QH-G0067	2015.001_20 Cannon	Other-source (not our collection)	The Morton Arboretum
SH-Q1151	QH-G0068	2015.003_9 Cannon	Other-source (not our collection)	The Morton Arboretum
SH-Q1152	QH-G0069	2015.001_21 Cannon	Other-source (not our collection)	The Morton Arboretum

SH-Q1153	QH-G0070	2015.003_10 Cannon	Other-source (not our collection)	The Morton Arboretum
SH-Q1154	QH-G0071	2015.001_22 Cannon	Other-source (not our collection)	The Morton Arboretum
SH-Q1155	QH-G0072	2016-0776	QH-E-1-4	Tulsa Botanic Garden
SH-Q1156	QH-G0073	2016-0777	QH-E-1-19	Tulsa Botanic Garden
SH-Q1157	QH-G0074	2016-0778	QH-E-1-21	Tulsa Botanic Garden
SH-Q1158	QH-G0075	2016-0779	QH-E-1-25	Tulsa Botanic Garden
SH-Q1159	QH-G0076	2016-0781	QH-E-4-12	Tulsa Botanic Garden
SH-Q1160	QH-G0077	2016-0782	QH-E-5-42	Tulsa Botanic Garden
SH-Q1161	QH-G0078	2016-0783	QH-E-7-5	Tulsa Botanic Garden
SH-Q1162	QH-G0079	2016-0785	QH-E-10-1	Tulsa Botanic Garden
SH-Q1163	QH-G0080	2016-0786	QH-E-10-7	Tulsa Botanic Garden
SH-Q1164	QH-G0081	2016-0787	QH-E-10-9	Tulsa Botanic Garden
SH-Q1165	QH-G0082	2016-0790	QH-E-AUX8-3	Tulsa Botanic Garden
SH-Q1166	QH-G0083	2016-0792	QH-W-3-1	Tulsa Botanic Garden
SH-Q1167	QH-G0084	436-2016sd	QH-E-AUX6-2	The Morton Arboretum
SH-Q1168	QH-G0085	436-2016sd	QH-E-AUX6-2	The Morton Arboretum
SH-Q1169	QH-G0086	432-2016sd	QH-E-AUX3	The Morton Arboretum
SH-Q1170	QH-G0087	432-2016sd	QH-E-AUX3	The Morton Arboretum
SH-Q1171	QH-G0088	451-2016sd	QH-W-AUX10-1	The Morton Arboretum
SH-Q1172	QH-G0089	451-2016sd	QH-W-AUX10-1	The Morton Arboretum
SH-Q1173	QH-G0090	451-2016sd	QH-W-AUX10-1	The Morton Arboretum
SH-Q1174	QH-G0091	451-2016sd	QH-W-AUX10-1	The Morton Arboretum
SH-Q1175	QH-G0092	451-2016sd	QH-W-AUX10-1	The Morton Arboretum
SH-Q1176	QH-G0093	451-2016sd	QH-W-AUX10-1	The Morton Arboretum
SH-Q1177	QH-G0094	451-2016sd	QH-W-AUX10-1	The Morton Arboretum
SH-Q1178	QH-G0095	451-2016sd	QH-W-AUX10-1	The Morton Arboretum
SH-Q1179	QH-G0096	451-2016sd	QH-W-AUX10-1	The Morton Arboretum
SH-Q1180	QH-G0097	451-2016sd	QH-W-AUX10-1	The Morton Arboretum
SH-Q1181	QH-G0098	451-2016sd	QH-W-AUX10-1	The Morton Arboretum
SH-Q1182	QH-G0099	451-2016sd	QH-W-AUX10-1	The Morton Arboretum
SH-Q1183	QH-G0100	451-2016sd	QH-W-AUX10-1	The Morton Arboretum
SH-Q1184	QH-G0101	451-2016sd	QH-W-AUX10-1	The Morton Arboretum
SH-Q1185	QH-G0102	451-2016sd	QH-W-AUX10-1	The Morton Arboretum
SH-Q1186	QH-G0103	451-2016sd	QH-W-AUX10-1	The Morton Arboretum
SH-Q1187	QH-G0104	451-2016sd	QH-W-AUX10-1	The Morton Arboretum
SH-Q1188	QH-G0105	451-2016sd	QH-W-AUX10-1	The Morton Arboretum
SH-Q1189	QH-G0106	451-2016sd	QH-W-AUX10-1	The Morton Arboretum
SH-Q1190	QH-G0107	451-2016sd	QH-W-AUX10-1	The Morton Arboretum

SH-Q1191	QH-G0108	443-2016sd	QH-E-4-9	The Morton Arboretum
SH-Q1192	QH-G0109	443-2016sd	QH-E-4-9	The Morton Arboretum
SH-Q1193	QH-G0110	448-2016sd	QH-E-1-19	The Morton Arboretum
SH-Q1194	QH-G0111	448-2016sd	QH-E-1-19	The Morton Arboretum
SH-Q1195	QH-G0112	428-2016sd	QH-E-11-1	The Morton Arboretum
SH-Q1196	QH-G0113	428-2016sd	QH-E-11-1	The Morton Arboretum
SH-Q1197	QH-G0114	428-2016sd	QH-E-11-1	The Morton Arboretum
SH-Q1198	QH-G0115	428-2016sd	QH-E-11-1	The Morton Arboretum
SH-Q1199	QH-G0116	430-2016sd	QH-E-12-1	The Morton Arboretum
SH-Q1200	QH-G0117	430-2016sd	QH-E-12-1	The Morton Arboretum
SH-Q1201	QH-G0118	430-2016sd	QH-E-12-1	The Morton Arboretum
SH-Q1202	QH-G0119	430-2016sd	QH-E-12-1	The Morton Arboretum
SH-Q1203	QH-G0120	430-2016sd	QH-E-12-1	The Morton Arboretum
SH-Q1204	QH-G0121	430-2016sd	QH-E-12-1	The Morton Arboretum
SH-Q1205	QH-G0122	431-2016sd	QH-E-8-19	The Morton Arboretum
SH-Q1206	QH-G0123	431-2016sd	QH-E-8-19	The Morton Arboretum
SH-Q1207	QH-G0124	431-2016sd	QH-E-8-19	The Morton Arboretum
SH-Q1208	QH-G0125	449-2016sd	QH-E-5-38	The Morton Arboretum
SH-Q1209	QH-G0126	449-2016sd	QH-E-5-38	The Morton Arboretum
SH-Q1210	QH-G0127	449-2016sd	QH-E-5-38	The Morton Arboretum
SH-Q1211	QH-G0128	450-2016sd	QH-E-1-31	The Morton Arboretum
SH-Q1212	QH-G0129	450-2016sd	QH-E-1-31	The Morton Arboretum
SH-Q1213	QH-G0130	450-2016sd	QH-E-1-31	The Morton Arboretum
SH-Q1214	QH-G0131	430-2016sd	QH-E-12-1	The Morton Arboretum
SH-Q1215	QH-G0132	450-2016sd	QH-E-1-31	The Morton Arboretum
SH-Q1216	QH-G0133	450-2016sd	QH-E-1-31	The Morton Arboretum
SH-Q1217	QH-G0134	450-2016sd	QH-E-1-31	The Morton Arboretum
SH-Q1218	QH-G0135	450-2016sd	QH-E-1-31	The Morton Arboretum
SH-Q1219	QH-G0136	450-2016sd	QH-E-1-31	The Morton Arboretum
SH-Q1220	QH-G0137	426-2016sd	QH-E-10-9	The Morton Arboretum
SH-Q1221	QH-G0138	426-2016sd	QH-E-10-9	The Morton Arboretum
SH-Q1222	QH-G0139	427-2016sd	QH-E-10-6	The Morton Arboretum
SH-Q1223	QH-G0140	427-2016sd	QH-E-10-6	The Morton Arboretum
SH-Q1224	QH-G0141	433-2016sd	QH-E-AUX4-2	The Morton Arboretum
SH-Q1225	QH-G0142	427-2016sd	QH-E-10-6	The Morton Arboretum
SH-Q1226	QH-G0143	427-2016sd	QH-E-10-6	The Morton Arboretum
SH-Q1227	QH-G0144	427-2016sd	QH-E-10-6	The Morton Arboretum
SH-Q1228	QH-G0145	442-2016sd	QH-E-6-1	The Morton Arboretum
SH-Q1229	QH-G0146	437-2016sd	QH-E-8-5	The Morton Arboretum

SH-Q1230	QH-G0147	437-2016sd	QH-E-8-5	The Morton Arboretum
SH-Q1231	QH-G0148	437-2016sd	QH-E-8-5	The Morton Arboretum
SH-Q1232	QH-G0149	437-2016sd	QH-E-8-5	The Morton Arboretum
SH-Q1233	QH-G0150	437-2016sd	QH-E-8-5	The Morton Arboretum
SH-Q1234	QH-G0151	441-2016sd	QH-E-5-23	The Morton Arboretum
SH-Q1235	QH-G0152	440-2016sd	QH-E-1-28	The Morton Arboretum
SH-Q1236	QH-G0153	440-2016sd	QH-E-1-28	The Morton Arboretum
SH-Q1237	QH-G0154	440-2016sd	QH-E-1-28	The Morton Arboretum
SH-Q1238	QH-G0155	440-2016sd	QH-E-1-28	The Morton Arboretum
SH-Q1239	QH-G0156	440-2016sd	QH-E-1-28	The Morton Arboretum
SH-Q1240	QH-G0157	513-2016sd	QH-W-3-13	The Morton Arboretum
SH-Q1241	QH-G0158	440-2016sd	QH-E-1-28	The Morton Arboretum
SH-Q1242	QH-G0159	440-2016sd	QH-E-1-28	The Morton Arboretum
SH-Q1243	QH-G0160	513-2016sd	QH-W-3-13	The Morton Arboretum
SH-Q1244	QH-G0161	513-2016sd	QH-W-3-13	The Morton Arboretum
SH-Q1245	QH-G0162	513-2016sd	QH-W-3-13	The Morton Arboretum
SH-Q1246	QH-G0163	446-2016sd	QH-E-3-25	The Morton Arboretum
SH-Q1247	QH-G0164	446-2016sd	QH-E-3-25	The Morton Arboretum
SH-Q1248	QH-G0165	QH-E-6-1.1	QH-E-6-1	Denver Botanical Garden
SH-Q1249	QH-G0166	QH-E-6-1.2	QH-E-6-1	Denver Botanical Garden
SH-Q1250	QH-G0167	QH-E-11-5.1	QH-E-11-5	Denver Botanical Garden
SH-Q1251	QH-G0168	QH-E-AuxZ.1	QH-E-AuxZ	Denver Botanical Garden
SH-Q1252	QH-G0169	QH-W-Aux10-3.1	QH-W-Aux10-3	Denver Botanical Garden
SH-Q1253	QH-G0170	QH-E-11-5.2	QH-E-11-5	Denver Botanical Garden
SH-Q1254	QH-G0171	QH-E-1-11.2	QH-E-1-11	Denver Botanical Garden
SH-Q1255	QH-G0172	QH-W-3-28.2	QH-W-3-28	Denver Botanical Garden
SH-Q1256	QH-G0173	QH-W-Aux3.2	QH-W-Aux3	Denver Botanical Garden
SH-Q1257	QH-G0174	QH-E-1-28.3	QH-E-1-28	Denver Botanical Garden
SH-Q1258	QH-G0175	QH-E-Aux4-Z.1	QH-E-Aux4-Z	Denver Botanical Garden
SH-Q1259	QH-G0176	QH-W-Aux3.1	QH-W-Aux3	Denver Botanical Garden
SH-Q1260	QH-G0177	QH-E-11-1.2	QH-E-11-1	Denver Botanical Garden
SH-Q1261	QH-G0178	QH-E-11-1.1	QH-E-11-1	Denver Botanical Garden
SH-Q1262	QH-G0179	QH-E-6-1.1	QH-E-6-1	Denver Botanical Garden
SH-Q1263	QH-G0180	QH-W-Aux3.3	QH-W-Aux3	Denver Botanical Garden
SH-Q1264	QH-G0181	QH-E-1-28.2	QH-E-1-28	Denver Botanical Garden
SH-Q1265	QH-G0182	QH-W-7-2.1	QH-W-7-2	Denver Botanical Garden
SH-Q1266	QH-G0183	QH-E-1-28.1	QH-E-1-28	Denver Botanical Garden
SH-Q1267	QH-G0184	QH-E-1-11.3	QH-E-1-11	Denver Botanical Garden
SH-Q1268	QH-G0185	QH-E-1-11.1	QH-E-1-11	Denver Botanical Garden

SH-Q1269	QH-G0186	QH-E-Aux8-1.1	QH-E-Aux8-1	Denver Botanical Garden
SH-Q1270	QH-G0187	QH-E-10-9	QH-E-10-9	Tulsa Botanic Garden
SH-Q1271	QH-G0188	QH-E-10-9	QH-E-10-9	Tulsa Botanic Garden
SH-Q1272	QH-G0189	QH-E-7-5	QH-E-7-5	Tulsa Botanic Garden
SH-Q1273	QH-G0190	QH-E-10-7	QH-E-10-7	Tulsa Botanic Garden
SH-Q1274	QH-G0191	QH-E-10-1	QH-E-10-1	Tulsa Botanic Garden
SH-Q1275	QH-G0192	QH-E-10-1	QH-E-10-1	Tulsa Botanic Garden
SH-Q1276	QH-G0193	QH-E-10-1	QH-E-10-1	Tulsa Botanic Garden
SH-Q1277	QH-G0194	QH-E-10-1	QH-E-10-1	Tulsa Botanic Garden
SH-Q1278	QH-G0195	QH-E-10-1	QH-E-10-1	Tulsa Botanic Garden
SH-Q1306	QH-G0196	QH-E-10-1	QH-E-10-1	Tulsa Botanic Garden
SH-Q1307	QH-G0197	QH-E-10-1	QH-E-10-1	Tulsa Botanic Garden
SH-Q1308	QH-G0198	QH-E-1-25	QH-E-1-25	Tulsa Botanic Garden
SH-Q1309	QH-G0199	QH-E-1-25	QH-E-1-25	Tulsa Botanic Garden
SH-Q1310	QH-G0200	QH-E-1-21	QH-E-1-21	Tulsa Botanic Garden
SH-Q1311	QH-G0201	QH-E-1-21	QH-E-1-21	Tulsa Botanic Garden
SH-Q1312	QH-G0202	QH-E-1-21	QH-E-1-21	Tulsa Botanic Garden
SH-Q1313	QH-G0203	QH-E-3-1	QH-E-3-1	Tulsa Botanic Garden
SH-Q1287	QH-G0204	QH-E-3-1	QH-E-3-1	Tulsa Botanic Garden
SH-Q1288	QH-G0205	QH-E-3-1	QH-E-3-1	Tulsa Botanic Garden
SH-Q1314	QH-G0206	QH-E-3-1	QH-E-3-1	Tulsa Botanic Garden
SH-Q1290	QH-G0207	QH-E-3-1	QH-E-3-1	Tulsa Botanic Garden
SH-Q1291	QH-G0208	QH-E-3-1	QH-E-3-1	Tulsa Botanic Garden
SH-Q1315	QH-G0209	QH-E-3-1	QH-E-3-1	Tulsa Botanic Garden
SH-Q1316	QH-G0210	QH-E-5-42	QH-E-5-42	Tulsa Botanic Garden
SH-Q1294	QH-G0211	QH-E-5-42	QH-E-5-42	Tulsa Botanic Garden
SH-Q1317	QH-G0212	QH-E-5-42	QH-E-5-42	Tulsa Botanic Garden
SH-Q1318	QH-G0213	QH-E-4-12	QH-E-4-12	Tulsa Botanic Garden
SH-Q1297	QH-G0214	QH-E-1-19	QH-E-1-19	Tulsa Botanic Garden
SH-Q1319	QH-G0215	QH-E-1-4	QH-E-1-4	Tulsa Botanic Garden
SH-Q1320	QH-G0216	QH-E-1-4	QH-E-1-4	Tulsa Botanic Garden
SH-Q1321	QH-G0217	QH-E-1-4	QH-E-1-4	Tulsa Botanic Garden
SH-Q1302	QH-G0218	QH-E-1-4	QH-E-1-4	Tulsa Botanic Garden
SH-Q1322	QH-G0219	QH-E-Aux8-3	QH-E-Aux8-3	Tulsa Botanic Garden
SH-Q1323	QH-G0220	QH-E-Aux8-3	QH-E-Aux8-3	Tulsa Botanic Garden
SH-Q1324	QH-G0221	QH-E-Aux8-3	QH-E-Aux8-3	Tulsa Botanic Garden
SH-Q1325	QH-G0222	QH-E-Aux8-3	QH-E-Aux8-3	Tulsa Botanic Garden
SH-Q1326	QH-G0223	5649-16P	QH-W-2-12	Chicago Botanic Garden
SH-Q1327	QH-G0224	5192-16P	QHE-Aux3	Chicago Botanic Garden

SH-Q1328	QH-G0225	5190-16P	QH-E-1-11	Chicago Botanic Garden
SH-Q1329	QH-G0226	5649-16P	QH-W-2-12	Chicago Botanic Garden
SH-Q1330	QH-G0227	5190-16P	QH-E-1-11	Chicago Botanic Garden
SH-Q1331	QH-G0228	5647-16P	QH-W-Aux3	Chicago Botanic Garden
SH-Q1332	QH-G0229	5647-16P	QH-W-Aux3	Chicago Botanic Garden
SH-Q1333	QH-G0230	5648-16P	QH-W-3-1	Chicago Botanic Garden
SH-Q1334	QH-G0231	5648-16P	QH-W-3-1	Chicago Botanic Garden
SH-Q1335	QH-G0232	5648-16P	QH-W-3-1	Chicago Botanic Garden
SH-Q1336	QH-G0233	5195-16P	QH-E Aux8-3	Chicago Botanic Garden
SH-Q1337	QH-G0234	5179-16P	QH-E-9	Chicago Botanic Garden
SH-Q1338	QH-G0235	5197-16P	QH-E Aux 6-1	Chicago Botanic Garden
SH-Q1339	QH-G0236	5197-16P	QH-E Aux 6-1	Chicago Botanic Garden
SH-Q1340	QH-G0237	5197-16P	QH-E Aux 6-1	Chicago Botanic Garden
SH-Q1341	QH-G0238	5197-16P	QH-E Aux 6-1	Chicago Botanic Garden
SH-Q1342	QH-G0239	5191-16P	QH-E-10-7	Chicago Botanic Garden
SH-Q1343	QH-G0240	5191-16P	QH-E-10-7	Chicago Botanic Garden
SH-Q1344	QH-G0241	5189-16P	QH-E-12-1	Chicago Botanic Garden
SH-Q1345	QH-G0242	5191-16P	QH-E-10-7	Chicago Botanic Garden
SH-Q1346	QH-G0243	5189-16P	QH-E-12-1	Chicago Botanic Garden
SH-Q1347	QH-G0244	5189-16P	QH-E-12-1	Chicago Botanic Garden
SH-Q1348	QH-G0245	5195-16P	QH-E Aux8-3	Chicago Botanic Garden
SH-Q1349	QH-G0246	5194-16P	QH-E Aux2	Chicago Botanic Garden
SH-Q1350	QH-G0247	5194-16P	QH-E Aux2	Chicago Botanic Garden
SH-Q1351	QH-G0248	5194-16P	QH-E Aux2	Chicago Botanic Garden
SH-Q1352	QH-G0249	5194-16P	QH-E Aux2	Chicago Botanic Garden
SH-Q1353	QH-G0250	5194-16P	QH-E Aux2	Chicago Botanic Garden
SH-Q1354	QH-G0251	5194-16P	QH-E Aux2	Chicago Botanic Garden
SH-Q1355	QH-G0252	5201-16P	QH-E-11-2	Chicago Botanic Garden
SH-Q1356	QH-G0253	5194-16P	QH-E Aux2	Chicago Botanic Garden
SH-Q1357	QH-G0254	5180-16P	QH-E-8-19	Chicago Botanic Garden
SH-Q1358	QH-G0255	5180-16P	QH-E-8-19	Chicago Botanic Garden
SH-Q1359	QH-G0256	5180-16P	QH-E-8-19	Chicago Botanic Garden
SH-Q1360	QH-G0257	5201-16P	QH-E-11-2	Chicago Botanic Garden
SH-Q1361	QH-G0258	5201-16P	QH-E-11-2	Chicago Botanic Garden
SH-Q1362	QH-G0259	5200-16P	QH-E-5-35	Chicago Botanic Garden
SH-Q1363	QH-G0260	5182-16P	QH-E-6-16	Chicago Botanic Garden
SH-Q1364	QH-G0261	5182-16P	QH-E-6-16	Chicago Botanic Garden
SH-Q1365	QH-G0262	5182-16P	QH-E-6-16	Chicago Botanic Garden
SH-Q1366	QH-G0263	5181-16P	QH-E-7-5	Chicago Botanic Garden

SH-Q1367	QH-G0264	5182-16P	QH-E-6-16	Chicago Botanic Garden
SH-Q1368	QH-G0265	5187-16P	QH-E-1-27	Chicago Botanic Garden
SH-Q1369	QH-G0266	5187-16P	QH-E-1-27	Chicago Botanic Garden
SH-Q1370	QH-G0267	5187-16P	QH-E-1-27	Chicago Botanic Garden
SH-Q1371	QH-G0268	5187-16P	QH-E-1-27	Chicago Botanic Garden
SH-Q1372	QH-G0269	5195-16P	QH-E Aux 8-3	Chicago Botanic Garden
SH-Q1373	QH-G0270	5195-16P	QH-E Aux 8-3	Chicago Botanic Garden
SH-Q1374	QH-G0271	5195-16P	QH-E Aux 8-3	Chicago Botanic Garden
SH-Q1375	QH-G0272	5181-16P	QH-E-7-5	Chicago Botanic Garden
SH-Q1376	QH-G0273	5181-16P	QH-E-7-5	Chicago Botanic Garden
SH-Q1377	QH-G0274	5181-16P	QH-E-7-5	Chicago Botanic Garden
SH-Q1378	QH-G0275	5199-16P	QH-E-Aux 4-1	Chicago Botanic Garden
SH-Q1379	QH-G0276	5199-16P	QH-E-Aux 4-1	Chicago Botanic Garden
SH-Q1380	QH-G0277	5199-16P	QH-E-Aux 4-1	Chicago Botanic Garden
SH-Q1381	QH-G0278	5181-16P	QH-E-7-5	Chicago Botanic Garden
SH-Q1382	QH-G0279	5199-16P	QH-E-Aux 4-1	Chicago Botanic Garden
SH-Q1383	QH-G0280	2018.047*1	QH-E-1-4 #2	Boyce Thompson Arboretum (BTA)
SH-Q1384	QH-G0281	2018.050*1	QH-E-1-21 #4	Boyce Thompson Arboretum (BTA)
SH-Q1385	QH-G0282	2018.051*1	QH-E-1-25 #2	Boyce Thompson Arboretum (BTA)
SH-Q1386	QH-G0283	2018.051*2	QH-E-1-25 #3	Boyce Thompson Arboretum (BTA)
SH-Q1387	QH-G0284	2018.049*1	QH-E-1-26 #3	Boyce Thompson Arboretum (BTA)
SH-Q1388	QH-G0285	2018.048*1	QH-E-5-42 #3	Boyce Thompson Arboretum (BTA)
SH-Q1389	QH-G0286	NA	QH-E-10-4	Private individual
SH-Q1390	QH-G0287	QH-E-6-16 (seed)	QH-E-6-16	Starhill Forest Arboretum
SH-Q1391	QH-G0288	QH-E-10-3 (seed)	QH-E-10-3	Starhill Forest Arboretum
SH-Q2011	QH-G0289	QUHA_E-7-5A	QH-E-7-5	Lady Bird Johnson
SH-Q2012	QH-G0290	QUHA_E-7-5B	QH-E-7-5	Lady Bird Johnson
SH-Q2013	QH-G0291	QUHA_E-7-5C	QH-E-7-5	Lady Bird Johnson
SH-Q2014	QH-G0292	QUHA_AUX8-3A	QUHA_AUX8-3	Lady Bird Johnson
SH-Q2015	QH-G0293	QUHA_AUX8-3B	QUHA_AUX8-3	Lady Bird Johnson
SH-Q2016	QH-G0294	QUHA_AUX8-3C	QUHA_AUX8-3	Lady Bird Johnson
SH-Q2017	QH-G0295	QUHA_E-1-31	QUHA_E-1-31	Lady Bird Johnson
SH-Q2018	QH-G0296	QUHA_WC2016-01	Other-source (not our collection)	Lady Bird Johnson
SH-Q2019	QH-G0297	QUHA_WC2016-02	Other-source (not our collection)	Lady Bird Johnson

Table S2. Forward and reverse sequence of each locus, repeat motif, multiplex groupings (Mpx), fluorescent dye used, the observed range of base pair numbers (size range), annealing temperature used in PCR reaction (T_a), and literature source.

Locus	Primer sequences (5′–3′)	Repeat motif	Mpx	Fluorescent dye	Size range (bp)	T_a (°C)	Source
MSQ4	F: TCTCCTCTCCCATAAACAGG<br>R: GTTCCTCTATCCAATCAGTAGTGAG	$(AG)_{17}$	2	VIC	178-248	50	Dow et al. 1995
MSQ13	F: TGGCTGCACCTATGGCTCTTAG<br>R: ACACTCAGACCCACCATTTTTCC	$(TC)_{14}$	1	6-FAM	190-244	54	Dow et al. 1995
QrZAG20	F: CCATTAAAAGAAGCAGTATTTTGT<br>R: GCAACACTCAGCCTATATCTAGAA	$(TC)_{18}$	1	VIC	155-189	50	Kampfer et al. 1998
QrZAG87	F: TCCCACCACTTTGGTCTCTCA<br>R: GTTGTCAGCAGTGGGATGGGTA	$(TC)_{20}$	3	NED	93-170	56	Kampfer et al. 1998
QpZAG110	F: GGAGGCTTCCTTCAACCTACT<br>R: GATCTCTTGTGTGCTGTATTT	$(AG)_{15}$	1	NED	161-243	50	Steinkellner et al. 1997
QpZAG1.5	F: GCTTGAGAGTTGAGATTTGT<br>R: GCAACACCCTTTAACTACCA	$(GT)_5(GA)_9$	3	PET	152-187	56	Steinkellner et al. 1997
QM69.2M1	F: CACAATCTGCCCACATCATC<br>R: GGATGGACGAAGAGAAAGAT	$(TGG)_6(CGG)\ (TGG)_2$	3	6-FAM	220-260	56	Isagi and Suhandono 1997
QS00314	F: TCAAAACGCAACGTTTCAAG<br>R: TTCGGGTTTTCTTTGTGGTC	$(GAA)_6$	1	PET	161-190	50	Chatwin et al. 2014
QS00562	F: ACCCCCACCTAATCCCAAC<br>R: TGCAAACACACAGAGACACTTTT	$(GA)_7$	3	6-FAM	214-282	56	Steinkellner et al 1997
QS01904	F: TCAGTCAAAAACCCACCTCC<br>R: GGGTTTTCTTCAGTTTGCTTGT	$(TC)_{10}$	2	6-FAM	131-155	54	Chatwin et al. 2014
QS03797	F: AGTTTTGGTTGATTGTGACGG<br>R: GCAAGGTCTGTCATTGGGTT	$(CA)_7$	1	6-FAM	140-169	50	Chatwin et al. 2014

Table S3. Results for the frequency of null alleles and a bootstrap confidence interval for each locus using the methods of Chakraborty et al. (1994) and Brookfield (1996). If the 95% confidence interval includes zero, it indicates that the frequency of null alleles at a locus does not significantly differ from zero.

		QS03797	MSQ13	QrZAG20	QpZAG110	QS00314	QS01904	MSQ4	QS00562	QrZAG87	QpZAG15	QM692M
Brookfield (1996)	Observed frequency	0.18	0.06	0.04	0.05	0.14	0.01	0.16	0.28	0.13	0.13	0.09
	Median frequency	0.18	0.05	0.04	0.05	0.14	0.01	0.16	0.29	0.13	0.13	0.09
	2.5th percentile	0.14	0.04	0.02	0.03	0.11	0	0.13	0.24	0.1	0.1	0.06
	97.5th percentile	0.21	0.08	0.06	0.07	0.17	0.03	0.19	0.33	0.15	0.16	0.11
Chakraborty et al. (1994)	Observed frequency	0.22	0.07	0.05	0.06	0.16	0.06	0.17	0.53	0.14	0.14	0.1
	Median frequency	0.22	0.07	0.05	0.06	0.16	0.05	0.17	0.54	0.14	0.14	0.1
	2.5th percentile	0.18	0.04	0.03	0.03	0.13	0	0.14	0.46	0.11	0.11	0.07
	97.5th percentile	0.27	0.09	0.07	0.09	0.2	0.13	0.2	0.62	0.17	0.17	0.12

Table S4. Results for each population for each locus for the following parameters: *N* = Number of individuals per population sample genotyped per locus, *A* = Total number of alleles observed per population sample per locus, % = Percentage of total alleles observed across population samples per population sample per locus, *Ar* = Allelic richness per locus, *Ho* = observed heterozygosity per locus, *He* = expected heterozygosity per locus, HWE = Hardy–Weinberg Equilibrium P-value from the X^2 goodness-of-fit tests per locus.

QH-E-1	QS03797	MSQ13	QrZAG20	QpZAG110	QS00314	QS01904	MSQ4	QS00562	QrZAG87	QpZAG15	QM692M	Overall
N	20	18	20	20	20	20	20	20	20	20	20	19.82
A	2	3	9	5	3	3	10	3	4	9	7	58
%	18.18	10.71	50	17.24	16.67	37.5	35.71	21.43	12.5	36	24.14	25.46
Ar	2	2.26	6.17	3.23	2.72	2.1	6.76	2.2	3.02	5.39	4.4	3.66
Ho	0.5	0.33	0.85	0.5	0.55	0.15	0.75	0.1	0.6	0.55	0.9	0.53
He	0.42	0.29	0.79	0.57	0.56	0.18	0.84	0.33	0.46	0.71	0.68	0.53
HWE	0.3929	0.8685	0.6733	0.0186	0.2328	0.03	0.0056	0	0.7212	0.202	0.8917	0.0013

QH-E-10	QS03797	MSQ13	QrZAG20	QpZAG110	QS00314	QS01904	MSQ4	QS00562	QrZAG87	QpZAG15	QM692M	Overall
N	9	6	9	8	8	9	8	9	9	9	9	8.45
A	4	4	9	4	4	3	8	3	4	8	4	55
%	36.36	14.29	50	13.79	22.22	37.5	28.57	21.43	12.5	32	13.79	25.68
Ar	3.82	2.84	7.01	3.24	3.9	2.24	5.86	2.49	3.49	5.9	3.21	4
Ho	0.67	0.5	1	0.62	0.75	0.22	0.62	0	0.78	0.56	0.67	0.58
He	0.73	0.42	0.85	0.58	0.73	0.2	0.84	0.49	0.56	0.81	0.5	0.61
HWE	0.1145	0.9951	0.4002	0.5235	0.3841	0.9866	0.5242	4.00E-04	0.7253	0.2123	0.8953	0.2524

QH-E-13	QS03797	MSQ13	QrZAG20	QpZAG110	QS00314	QS01904	MSQ4	QS00562	QrZAG87	QpZAG15	QM692M	Overall
N	8	8	8	8	8	8	8	8	8	8	8	8
A	3	6	8	5	4	2	7	2	5	5	6	53
%	27.27	21.43	44.44	17.24	22.22	25	25	14.29	15.62	20	20.69	23.02
Ar	2.65	4.6	6.58	4.3	3.44	1.66	5.39	1.9	4.17	3.96	5.49	4.01
Ho	0.62	0.75	0.88	0.88	0.5	0.12	0.62	0.25	0.75	0.75	1	0.65
He	0.55	0.67	0.83	0.73	0.49	0.12	0.78	0.22	0.69	0.64	0.81	0.59
HWE	0.68	0.9238	0.2256	0.2693	0.2478	0.8415	0.5213	0.6892	0.4872	0.971	0.6623	0.7722

Table S4. Continued.

QH-E-14	QS03797	MSQ13	QrZAG20	QpZAG110	QS00314	QS01904	MSQ4	QS00562	QrZAG87	QpZAG15	QM692M	Overall
N	21	20	24	24	24	24	24	23	24	24	24	23.27
A	5	8	10	7	9	4	13	2	7	8	11	84
%	45.45	28.57	55.56	24.14	50	50	46.43	14.29	21.88	32	37.93	36.93
Ar	3.63	4.84	6.92	4.8	5.55	2.6	7.13	1.89	4.19	5.79	6.76	4.92
Ho	0.52	0.75	0.96	0.58	0.67	0.21	0.75	0.13	0.75	0.46	0.79	0.6
He	0.67	0.7	0.85	0.7	0.78	0.32	0.85	0.31	0.67	0.84	0.83	0.69
HWE	0.1996	1.00E-04	0.7156	0.2225	0	0.1513	4.00E-04	0.005	1.00E-04	0	0.4994	0

QH-E-2	QS03797	MSQ13	QrZAG20	QpZAG110	QS00314	QS01904	MSQ4	QS00562	QrZAG87	QpZAG15	QM692M	Overall
N	25	25	25	25	25	25	25	25	25	25	25	25
A	3	3	5	8	3	3	13	2	3	6	7	56
%	27.27	10.71	27.78	27.59	16.67	37.5	46.43	14.29	9.38	24	24.14	24.16
Ar	2.94	2.23	3.94	4.56	2.85	2.25	6	2	2.31	4.49	4.46	3.46
Ho	0.44	0.24	0.6	0.72	0.56	0.24	0.56	0.32	0.52	0.56	0.6	0.49
He	0.6	0.22	0.67	0.64	0.52	0.22	0.77	0.5	0.39	0.71	0.72	0.54
HWE	0.1785	0.9276	0.392	0.9949	0.334	0.9276	7.00E-04	0.0727	0.378	0.0056	0.9007	0.0183

QH-E-3	QS03797	MSQ13	QrZAG20	QpZAG110	QS00314	QS01904	MSQ4	QS00562	QrZAG87	QpZAG15	QM692M	Overall
N	27	27	27	27	27	27	27	27	27	27	27	27
A	4	2	8	7	3	3	11	2	4	8	5	57
%	36.36	7.14	44.44	24.14	16.67	37.5	39.29	14.29	12.5	32	17.24	25.6
Ar	3.37	1.76	4.99	3.83	2.23	1.74	6.39	1.93	2.51	4.14	3.45	3.3
Ho	0.67	0.15	0.63	0.48	0.44	0.11	0.48	0.22	0.41	0.41	0.56	0.41
He	0.59	0.14	0.65	0.66	0.52	0.11	0.85	0.3	0.36	0.58	0.65	0.49
HWE	0.5323	0.6801	7.00E-04	0.0032	0.6708	0.993	1.00E-04	0.1703	0.4494	0.047	1.00E-04	0

Table S4. Continued.

QH-E-4	QS03797	MSQ13	QrZAG20	QpZAG110	QS00314	QS01904	MSQ4	QS00562	QrZAG87	QpZAG15	QM692M	Overall
N	19	19	17	19	16	19	16	19	19	19	18	18.18
A	3	3	10	4	3	2	6	2	4	6	6	49
%	27.27	10.71	55.56	13.79	16.67	25	21.43	14.29	12.5	24	20.69	21.99
Ar	2.93	2.26	6.14	3.15	2.32	1.92	4.38	1.59	3.42	4.43	4.25	3.34
Ho	0.47	0.32	0.76	0.63	0.44	0.26	0.5	0.05	0.63	0.37	0.78	0.47
He	0.59	0.27	0.82	0.51	0.42	0.23	0.76	0.15	0.55	0.76	0.69	0.52
HWE	0.2261	0.8802	0.0416	0.7199	0.9402	0.5071	0.3189	0.0054	0.4356	0	0.8817	0.0012

QH-E-5	QS03797	MSQ13	QrZAG20	QpZAG110	QS00314	QS01904	MSQ4	QS00562	QrZAG87	QpZAG15	QM692M	Overall
N	32	31	32	32	32	32	32	32	32	32	32	31.91
A	3	4	7	4	6	2	9	3	5	9	8	60
%	27.27	14.29	38.89	13.79	33.33	25	32.14	21.43	15.62	36	27.59	25.94
Ar	2.39	2.96	4.41	3.25	2.91	2	4.93	1.73	2.91	4.56	4.59	3.33
Ho	0.28	0.55	0.78	0.62	0.47	0.62	0.75	0.03	0.69	0.28	0.62	0.52
He	0.25	0.46	0.67	0.6	0.4	0.43	0.7	0.2	0.54	0.66	0.58	0.5
HWE	0.8351	0.3968	0.9191	0.1906	0	0.0101	0.9771	0	0.6978	0	0.8017	0

QH-E-6	QS03797	MSQ13	QrZAG20	QpZAG110	QS00314	QS01904	MSQ4	QS00562	QrZAG87	QpZAG15	QM692M	Overall
N	14	14	14	14	14	14	14	14	14	14	14	14
A	3	6	8	7	5	2	8	5	4	7	5	60
%	27.27	21.43	44.44	24.14	27.78	25	28.57	35.71	12.5	28	17.24	26.55
Ar	2.82	4.3	6.38	5.22	4.14	1.72	5.79	3.61	3.16	5.68	3.64	4.22
Ho	0.5	0.5	0.93	0.93	0.5	0.14	0.79	0.36	0.5	0.57	0.57	0.57
He	0.48	0.61	0.82	0.74	0.68	0.13	0.79	0.68	0.49	0.82	0.62	0.63
HWE	0.6846	0.4536	0.4955	0.3629	0.2198	0.7773	0.0097	3.00E-04	0.2142	0.0718	0.7965	0.0026

Table S4 Continued.

QH-E-7	QS03797	MSQ13	QrZAG20	QpZAG110	QS00314	QS01904	MSQ4	QS00562	QrZAG87	QpZAG15	QM692M	Overall
N	56	51	56	55	56	56	56	56	56	56	56	55.45
A	5	9	10	9	8	5	13	6	4	13	9	91
%	45.45	32.14	55.56	31.03	44.44	62.5	46.43	42.86	12.5	52	31.03	41.45
Ar	3.9	3.12	5.41	4.32	3.6	2.4	7.21	2.65	3.14	6.12	4.03	4.17
Ho	0.55	0.47	0.71	0.69	0.64	0.2	0.73	0.23	0.48	0.52	0.66	0.54
He	0.69	0.48	0.69	0.69	0.58	0.21	0.86	0.36	0.5	0.83	0.7	0.6
HWE	0.0249	0.0063	0	1.00E-04	0	0.8622	0	0	2.00E-04	0	0.0011	0

QH-E-AUX5	QS03797	MSQ13	QrZAG20	QpZAG110	QS00314	QS01904	MSQ4	QS00562	QrZAG87	QpZAG15	QM692M	Overall
N	11	11	11	11	11	11	11	11	11	11	11	11
A	3	3	2	2	2	1	6	2	3	5	5	34
%	27.27	10.71	11.11	6.9	11.11	12.5	21.43	14.29	9.38	20	17.24	14.72
Ar	2.52	2.32	1.98	2	1.99	1	5.21	1.99	2.61	4.38	4.39	2.76
Ho	0.64	0.09	0.36	0.36	0.45	0	0.45	0.27	0.18	0.36	1	0.38
He	0.46	0.37	0.3	0.46	0.35	0	0.81	0.43	0.31	0.69	0.71	0.45
HWE	0.4936	0.0115	0.4624	0.4751	0.3297	NA	1.00E-04	0.2176	1.00E-04	2.00E-04	0.1828	0

QH-W-1	QS03797	MSQ13	QrZAG20	QpZAG110	QS00314	QS01904	MSQ4	QS00562	QrZAG87	QpZAG15	QM692M	Overall
N	29	39	39	39	39	39	39	39	39	39	39	38.09
A	3	7	5	10	3	2	8	4	5	7	4	58
%	27.27	25	27.78	34.48	16.67	25	28.57	28.57	15.62	28	13.79	24.61
Ar	1.96	3.98	3.48	5.74	2.18	1.17	5.16	2.59	2.05	5.19	3.12	3.33
Ho	0.03	0.72	0.72	0.85	0.38	0.03	0.67	0.15	0.13	0.79	0.64	0.46
He	0.39	0.67	0.62	0.79	0.51	0.03	0.78	0.5	0.17	0.77	0.61	0.53
HWE	0	0.1546	1.00E-04	7.00E-04	0.2957	0.9203	0.1032	0	0.5474	0.0027	0.7975	0

Table S4. Continued.

QH-W-10	QS03797	MSQ13	QrZAG20	QpZAG110	QS00314	QS01904	MSQ4	QS00562	QrZAG87	QpZAG15	QM692M	Overall
N	29	29	29	29	29	29	29	29	27	29	29	28.82
A	3	8	11	7	5	4	12	6	11	13	10	90
%	27.27	28.57	61.11	24.14	27.78	50	42.86	42.86	34.38	52	34.48	38.68
Ar	2.96	5.11	6.79	5.18	3.27	1.75	7.43	3.56	6.98	8.15	5.43	5.15
Ho	0.38	0.69	0.9	0.69	0.41	0.1	0.76	0.1	0.96	0.79	0.72	0.59
He	0.65	0.72	0.85	0.77	0.45	0.1	0.87	0.6	0.86	0.89	0.63	0.67
HWE	0.0015	0.8699	0.7098	0	6.00E-04	1	0.001	0	0.0489	0.0127	0.5407	0

QH-W-11	QS03797	MSQ13	QrZAG20	QpZAG110	QS00314	QS01904	MSQ4	QS00562	QrZAG87	QpZAG15	QM692M	Overall
N	20	20	20	20	20	20	20	20	20	20	20	20
A	6	11	8	7	6	1	15	6	7	9	8	84
%	54.55	39.29	44.44	24.14	33.33	12.5	53.57	42.86	21.88	36	27.59	35.47
Ar	3.8	7.55	5.84	4.32	5.26	1	8.75	3.75	3.49	5.4	5.49	4.97
Ho	0.25	0.9	0.8	0.8	0.75	0	0.8	0.35	0.35	0.6	0.85	0.59
He	0.63	0.88	0.77	0.69	0.8	0	0.89	0.46	0.35	0.66	0.78	0.63
HWE	0.0019	0.1637	0.1406	0.7965	0.232	NA	0.0219	0	0.9015	0.0569	0.5014	0

QH-W-12	QS03797	MSQ13	QrZAG20	QpZAG110	QS00314	QS01904	MSQ4	QS00562	QrZAG87	QpZAG15	QM692M	Overall
N	18	18	18	18	18	18	18	18	18	17	18	17.91
A	2	12	8	7	6	1	12	2	6	9	4	69
%	18.18	42.86	44.44	24.14	33.33	12.5	42.86	14.29	18.75	36	13.79	27.38
Ar	1.97	7.7	5.65	4.73	4.61	1	7.33	1.37	4.12	5.23	3.15	4.26
Ho	0	0.94	0.61	0.61	0.56	0	0.72	0	0.44	0.71	0.39	0.45
He	0.44	0.88	0.82	0.62	0.78	0	0.86	0.1	0.5	0.73	0.57	0.57
HWE	0	0.1153	0.0083	0.1715	0	NA	1.00E-04	0	0.0018	0.0012	0.0159	0

Table S4. Continued.

QH-W-2	QS03797	MSQ13	QrZAG20	QpZAG110	QS00314	QS01904	MSQ4	QS00562	QrZAG87	QpZAG15	QM692M	Overall
N	11	11	11	11	11	11	11	11	11	11	11	11
A	3	6	6	4	5	1	5	2	9	7	5	53
%	27.27	21.43	33.33	13.79	27.78	12.5	17.86	14.29	28.12	28	17.24	21.96
Ar	2.93	5.28	4.82	3.57	3.69	1	3.78	2	7.26	5.41	4.25	4
Ho	0.27	0.73	0.55	0.73	0.45	0	0	0.64	0.91	0.82	0.36	0.5
He	0.66	0.79	0.74	0.55	0.51	0	0.74	0.5	0.87	0.8	0.75	0.63
HWE	0.0315	0.0508	0.0019	0.732	0.2706	NA	0	0.3652	0.007	0.0023	0.0347	0

QH-W-3	QS03797	MSQ13	QrZAG20	QpZAG110	QS00314	QS01904	MSQ4	QS00562	QrZAG87	QpZAG15	QM692M	Overall
N	28	33	33	33	32	33	33	33	33	33	33	32.45
A	5	12	8	9	6	3	13	4	10	12	6	88
%	45.45	42.86	44.44	31.03	33.33	37.5	46.43	28.57	31.25	48	20.69	37.23
Ar	2.83	7.1	5.42	4.61	3.42	1.59	7.53	1.82	6.45	6.99	3.64	4.67
Ho	0.25	0.91	0.7	0.42	0.47	0.09	0.52	0.03	0.79	0.67	0.52	0.49
He	0.39	0.86	0.74	0.59	0.49	0.09	0.9	0.17	0.78	0.85	0.45	0.57
HWE	0.1034	0.3008	0.2481	0.0013	0.5876	0.9952	0	0	0.3696	0.48	0.999	0
Fis	0.3595	-0.0622	0.0641	0.2804	0.0457	-0.0366	0.4273	0.8221	-0.0041	0.2181	-0.1461	0.1521

QH-W-4	QS03797	MSQ13	QrZAG20	QpZAG110	QS00314	QS01904	MSQ4	QS00562	QrZAG87	QpZAG15	QM692M	Overall
N	24	33	33	33	33	33	33	32	25	33	33	31.36
A	4	12	9	5	8	2	10	6	8	7	8	79
%	36.36	42.86	50	17.24	44.44	25	35.71	42.86	25	28	27.59	34.1
Ar	3.01	6.66	6.3	3.29	4.85	1.9	6.58	3.33	4.51	4.82	4.65	4.54
Ho	0.38	1	0.94	0.52	0.79	0.27	0.82	0.09	0.6	0.79	0.73	0.63
He	0.47	0.82	0.83	0.59	0.75	0.24	0.84	0.61	0.71	0.66	0.64	0.65
HWE	0.1338	0.3066	0.0182	0.8885	0.0095	0.3652	0	0	0.2966	0.9036	0.8498	0

Table S4. Continued.

QH-W-5	QS03797	MSQ13	QrZAG20	QpZAG110	QS00314	QS01904	MSQ4	QS00562	QrZAG87	QpZAG15	QM692M	Overall
N	30	30	30	30	30	30	30	29	30	29	30	29.82
A	4	13	12	10	7	3	15	3	11	16	9	103
%	36.36	46.43	66.67	34.48	38.89	37.5	53.57	21.43	34.38	64	31.03	42.25
Ar	3.65	6.42	7.02	4.87	4.23	1.96	7.85	1.82	5.94	8.64	6.33	5.34
Ho	0.63	0.73	0.83	0.63	0.7	0.17	0.73	0.03	0.73	0.86	0.9	0.63
He	0.7	0.76	0.86	0.6	0.68	0.16	0.87	0.22	0.7	0.9	0.82	0.66
HWE	0.0028	0.7443	0.4796	0.9196	0	0.9691	0	0	0.9592	0.0175	0.1769	0

QH-W-6	QS03797	MSQ13	QrZAG20	QpZAG110	QS00314	QS01904	MSQ4	QS00562	QrZAG87	QpZAG15	QM692M	Overall
N	33	33	33	33	33	33	33	33	33	33	33	33
A	4	12	14	8	5	3	18	4	14	14	10	106
%	36.36	42.86	77.78	27.59	27.78	37.5	64.29	28.57	43.75	56	34.48	43.36
Ar	3.49	6.12	7.47	4.08	3.25	1.93	8.4	2.91	6.93	7.41	4.72	5.16
Ho	0.36	0.79	0.82	0.55	0.52	0.15	0.67	0.12	0.7	0.73	0.58	0.54
He	0.71	0.73	0.85	0.57	0.52	0.14	0.89	0.51	0.79	0.84	0.58	0.65
HWE	0	0.9763	0.7893	0.9986	0.777	0.9743	0.012	0	0.1228	3.00E-04	0	0

QH-W-7	QS03797	MSQ13	QrZAG20	QpZAG110	QS00314	QS01904	MSQ4	QS00562	QrZAG87	QpZAG15	QM692M	Overall
N	32	32	32	32	32	32	32	32	32	32	32	32
A	4	9	14	10	9	1	14	5	11	7	9	93
%	36.36	32.14	77.78	34.48	50	12.5	50	35.71	34.38	28	31.03	38.4
Ar	3.64	5.81	7.63	5.17	5.42	1	6.67	2.84	6.58	5.16	4.93	4.99
Ho	0.44	0.62	0.91	0.78	0.75	0	0.53	0.09	0.75	0.72	0.66	0.57
He	0.66	0.71	0.87	0.7	0.75	0	0.83	0.47	0.83	0.75	0.6	0.65
HWE	0.0121	0.649	0.3672	0.2292	0.2839	NA	0	0	0.8163	0.5922	0.0832	0

Table S4. Continued.

QH-W-8	QS03797	MSQ13	QrZAG20	QpZAG110	QS00314	QS01904	MSQ4	QS00562	QrZAG87	QpZAG15	QM692M	Overall
N	39	39	39	39	39	39	39	37	38	39	39	38.73
A	4	9	10	8	6	4	17	6	12	15	10	101
%	36.36	32.14	55.56	27.59	33.33	50	60.71	42.86	37.5	60	34.48	42.78
Ar	3.15	6.31	6.35	4.18	3.36	2.55	8.3	3.62	6.13	7.39	5.28	5.15
Ho	0.51	0.9	0.85	0.51	0.69	0.26	0.74	0.16	0.71	0.74	0.74	0.62
He	0.66	0.83	0.83	0.58	0.63	0.31	0.9	0.62	0.78	0.86	0.69	0.7
HWE	0.004	0	0.6219	4.00E-04	0	0.4288	0.2433	0	0.7631	0.0701	0.8293	0

QH-W-9	QS03797	MSQ13	QrZAG20	QpZAG110	QS00314	QS01904	MSQ4	QS00562	QrZAG87	QpZAG15	QM692M	Overall
N	30	30	30	30	30	30	30	29	30	30	30	29.91
A	3	13	13	10	7	3	13	5	13	15	11	106
%	27.27	46.43	72.22	34.48	38.89	37.5	46.43	35.71	40.62	60	37.93	43.41
Ar	2.97	6.56	7.97	4.82	4.13	1.49	7.46	3.19	7.9	7	6.05	5.41
Ho	0.43	0.77	0.87	0.5	0.5	0.03	0.67	0.07	0.87	0.77	0.67	0.56
He	0.65	0.77	0.88	0.63	0.6	0.1	0.88	0.57	0.88	0.84	0.74	0.68
HWE	0.001	6.00E-04	0.0057	0.0481	0.1084	0	0	0	0.3774	0.5325	0.0474	0

QH-W-AUX3	QS03797	MSQ13	QrZAG20	QpZAG110	QS00314	QS01904	MSQ4	QS00562	QrZAG87	QpZAG15	QM692M	Overall
N	16	15	16	15	16	16	16	16	9	16	16	15.18
A	3	7	12	7	6	2	9	6	5	9	7	73
%	27.27	25	66.67	24.14	33.33	25	32.14	42.86	15.62	36	24.14	32.02
Ar	2.98	5.73	8.08	4.53	4.1	1.43	6.38	3.85	3.82	6.02	4.91	4.71
Ho	0.62	0.93	1	0.67	0.62	0.06	0.62	0.12	0.89	0.81	0.75	0.65
He	0.64	0.8	0.88	0.68	0.64	0.06	0.84	0.7	0.69	0.77	0.62	0.66
HWE	0.0087	0.649	0.6092	0.7409	0.7252	0.8875	9.00E-04	0	0.8059	0.1004	0.9987	0.013

Table S5. Results from the full dataset. Percent alleles captured by each garden for each allele category, and the number of plants (num ind ex situ), number of maternal accessions sampled (num mat plants) and number of populations sampled (num pops), and number of populations from East and West regions.

garden	global	Global common	Glob low frequency	Global rare	num ind ex situ	num mat plants	num pops	East pops	West pops
A	8	35	5	0	1	1	1	1	0
B	11	41	15	0	2	2	2	2	0
C	16	53	21	2	6	2	6	2	0
D	21	69	30	4	9	3	3	3	0
E	36	94	57	10	22	10	12	6	4
F	37	92	61	9	43	8	14	7	1
G	43	96	70	14	56	18	19	15	3
H	65	100	89	42	151	20	45	15	5
all	70	100	94	48	290	26	87	18	8

Table S6. Results from the reduced dataset. Percent alleles captured by each garden for each allele category, and the number of plants (num ind ex situ), number of maternal accessions sampled (num mat plants) and number of populations sampled (num pops), and number of populations from East and West regions.

garden	global	Global common	Glob low frequency	Global rare	num ind ex situ	num mat plants	num pops	East pops	West pops
A	10	35	5	0	1	1	1	1	0
B	14	41	15	0	2	2	2	2	0
C	20	53	21	1	6	2	6	2	0
D	26	69	30	3	9	3	3	3	0
E	46	94	57	13	22	10	12	6	4
F	47	92	61	13	43	8	14	7	1
G	52	96	70	14	56	18	19	15	3
H	76	100	89	51	151	20	45	15	5
all	79	100	94	55	290	26	87	18	8

Methods S1. Modified DNA extraction protocol using the E.Z.N.A. Plant DNA DS kits (Omega Bio-tek, Inc., Norcross, GA, USA).

1) **Prep Work**:
☐ Wipe down area/gloves with 10% bleach and 70% ethanol in between samples to avoid cross contamination
☐ Turn on 65°C hot water bath & add bottle of elution buffer
☐ Wash work area, wipe down centrifuges, FastPrep, as well as tweezers and spatulas with ETOH or 10% bleach
☐ Label 2 tubes (1 x 1.5mL tube (For final storage), 1 x 2mL tube) and green spin column for each sample
☐ Label 1 FastPrep tube for each sample. Add 6 stainless steel beads to each FastPrep tube
☐ Get cooler of ice from basement

2) ☐ For every sample, measure **0.035 - 0.060 g** of leaf tissue and Transfer into FastPrep tube. Put FastPrep tube on ice. Tubes may also be placed in -20C freezer as they are filled to reduce thawing of leaves. Repeat for all samples. **Use forceps/scissors to hold leaf to avoid defrosting leaf with heat of hand. Make sure to clean forceps with 10% bleach between each sample.

3) ☐ Place all tubes in the -80 °C freezer for at least 30 minutes. Usually 60 minutes is optimal. The tubes can remain in the freezer for longer than 30 minutes, even overnight if necessary. *In Time*:__________ *Out Time*:__________

4) ☐ After freezer incubation period, add 700µL **CSPL buffer** and 20µL **Proteinase K Solution**

5) ☐ Place the tubes in the FastPrep rotor, twist the rotor so the spokes cover the tubes, place the rotor back in the FastPrep machine, spin the rotor clockwise until it clicks into place, secure the rotor with the securing knob, ratchet the securing knob to ensure rotor is stable, and close the dome lid, run the auto-program "quickprep 2x30" which is two cycles of 6.0m/s for 30 seconds.

6) ☐ Incubate entire batch for 30 - 60 min. at 65°C, inverting tubes 2-3 times. I*n Time*:__________ *Out Time*:__________

7) ☐ Spin for *3 minutes at 12,000 x g.*

8) ☐ Apply 550µL lysate to Homogenizer spin column (green) sitting in a 2ml collection tube
☐ Centrifuge homogenizer columns for 1 min. at 12,000 x g

9) ☐ Throw away top green portion of tube, **keep lower portion**.
☐ Transfer flow-through fraction (lower portion!) to labeled 2ml tube without disturbing the cell debris pellet

10) ☐ Add **5uL RNase A** and gently invert or lightly vortex. Let sit at room temperature for 5 minutes *In Time*:__________ *Out Time*:__________

11) ☐ Add **525uL RBB buffer** and **525uL XP2 buffer**. Vortex to mix thoroughly

12) ☐☐ Apply 750µl of this mixture, including any precipitate, onto HiBind mini spin column (blue) sitting in a 2ml collection tube (Can dump into spin column second time)
☐☐ Centrifuge *for 1 min. at 12,000 x g*
☐☐ Pour out lower portion, **keep blue column**
****Repeat step 12 ****

13) ☐ Add **500uL HBC buffer**

14) ☐ Centrifuge at 12,000 x g for 1 minute

15) ☐ Pour out lower portion, **keep blue column**

16) ☐☐ Add **700uL DNA Wash buffer**
☐☐ Centrifuge *for 1 min. at 12,000 x g*
☐☐ Pour out lower portion,**keep blue column**
****Repeat step 16 ****

17) ☐ Centrifuge column for 2 minutes at 12,000 x g

18) ☐ Transfer column into 1.5ml tube with caps ripped off

19) ☐☐ Pipet **55uL** of preheated **elution buffer** onto column membrane. *In Time*:__________ *Out Time*:__________
☐☐ Incubate for 10 min. at room temperature *In Time*:________*Out Time*:__________
☐☐ Centrifuge for 1 min. @ 12,000 x g to elute (lower portion contains DNA!) Make sure to use "clean" centrifuge at end of lab bench
****Repeat step 19 ****

20) ☐ Transfer flow through to a labeled 1.5mL tube for long term storage

Clean-Up:

21) ☐ Add DNA specimen information to DNA Database/Put paperwork in extractions binder
☐ Store DNA in **-20 °C** freezer temporarily or **-80 °C** freezer for long term
☐ Turn off hot water bath and clean all equipment (centrifuges, FastPrep, pipettes, etc.) with 10% bleach followed by water
☐ Clean beads in 10% bleach solution and rinse with distilled H2O.
☐ Quantify DNA & add this information to our DNA database

References

Agapow, P.-M., & Burt, A. (2001). Indices of multilocus linkage disequilibrium. *Molecular ecology notes*, *1*, 101-102. doi: 10.1046/j.1471-8278.2000.00014.x

Brookfield, J. F. Y. (1996). A simple new method for estimating null allele frequency from heterozygote deficiency. *Molecular ecology*, *5*(3), 453-455. doi:10.1046/j.1365-294X.1996.00098.x

Chakraborty, R., Andrade, M. D., Daiger, S. P., & Budowle, B. (1992). Apparent heterozygote deficiencies observed in DNA typing data and their implications in forensic applications. *Annals of human genetics*, *56*(1), 45-57.

Chatwin, W. B., Carpenter, K. K., Jimenez, F. R., Elzinga, D. B., Johnson, L. A., & Maughan, P. J. (2014). Microsatellite primer development for post oak, *Quercus stellata* (Fagaceae). *Applications in plant sciences*, *2*(10), 1400070. doi: 10.3732/apps.1400070

Dow, B. D., Ashley, M. V., & Howe, H. F. (1995). Characterization of highly variable (GA/CT) n microsatellites in the bur oak, *Quercus macrocarpa*. *Theoretical and applied genetics*, *91*(1), 137-141. doi: 10.1007/BF00220870

Hoban, S., & Schlarbaum, S. (2014). Optimal sampling of seeds from plant populations for ex-situ conservation of genetic biodiversity, considering realistic population structure. *Biological Conservation*, *177*, 90–99. doi: 10.1016/j.biocon.2014.06.014

Isagi, Y., & Suhandono, S. (1997). PCR primers amplifying microsatellite loci of *Quercus myrsinifolia* Blume and their conservation between oak species. *Molecular ecology*, *6*(9), 897-899. doi: 10.1111/j.1365-294x.1997.tb00147.x

Kampfer, S., Lexer, C., Glossl, J., & Steinkellner, H. (1998). Brief report characterization of (GA) n microsatellite loci from *Quercus robur*. *Hereditas*, *129*(183), 1-86. doi:
https://doi.org/10.1111/j.1601-5223.1998.00183.x

Steinkellner, H., Lexer, C., Turetschek, E., & Glössl, J. (1997). Conservation of (GA) n microsatellite loci between *Quercus* species. *Molecular ecology*, *6*(12), 1189-1194. doi: 10.1046/j.1365-294X.1997.00288.x
